# Impact of a Mixed Ocean Layer and the Diurnal Cycle on Convective Aggregation

**DOI:** 10.1029/2020MS002186

**Published:** 2021-11-25

**Authors:** Adrian M. Tompkins, Addisu G. Semie

**Affiliations:** ^1^ Earth System Physics Abdus Salam International Centre for Theoretical Physics (ICTP) Trieste Italy; ^2^ Laboratoire de Meteorologie Dynamique (LMD/IPSL) Sorbonne University CNRS Paris France; ^3^ Computational Data Science Program Addis Ababa University Addis Ababa Ethiopia

**Keywords:** Convection, organization, radiative, equilibrium, tropics, clouds

## Abstract

We investigate how ocean feedbacks and the diurnal cycle impact convective aggregation using a slab ocean coupled to a cloud resolving model. With a 20 m mixed layer ocean, aggregation occurs after 25 days. Thinner ocean layers slow the onset of clustering, with a 1 m ocean layer needing around 43 days. The delay is due to anomalous solar radiation in clear sky regions, causing a relative surface warming balanced by a matching cooling in cloudy areas. The resulting gradient in surface heat fluxes opposes low level convergence into convecting regions. On aggregation, the convective regions are surrounded by moist, clear sky regions with the hottest SSTs, toward which convection migrates, while a cold SST patch forms under dry suppressed regions due to enhanced latent heat fluxes and longwave radiation. The next experiment allows a diurnal cycle of 2.5°C in the domain mean SST. This causes convective rainfall to shift from a weak morning maximum to a sharper evening peak, reminiscent of undisturbed tropical observations. However, convection reverts to a weak early morning maximum once aggregation starts due to spatially heterogeneous radiative forcing. This implies thin mixed ocean layers are a necessary but non‐sufficient condition for an afternoon maximum of convection; limited spatial water vapor variability is also necessary. The imposition of the mean diurnal cycle has no statistically significant impact on the mean timing of clustering onset.

## Key Points

- Interactive SSTs acts to slow the onset of convective aggregation, via shortwave cloud forcing and latent heat fluxes
- Slower aggregation onset is also highly variable, as expected for a weakly forced, bi‐stable stochastic system
- Allowing a diurnal cycle in the mean surface temperature has no significant impact on aggregation timing

## 1 Introduction

Over two decades ago, the first month‐long idealized studies of radiative‐convective equilibrium using kilometer scale, convection‐permitting resolutions demonstrated that convection may sometimes (but not always) spontaneously organize into convective clusters (Held et al., 1993; Tompkins & Craig, 1998a). In the subsequent period, various simulations have demonstrated some of the diabatic feedbacks that could lead to this organization, including surface flux feedback (Tompkins & Craig, 1998a; Wing & Emanuel, 2014), water vapor feedbacks (Grabowski & Moncrieff, 2004; Held et al., 1993; Tompkins, 2001b), and radiative feedbacks involving water vapor and clouds (Emanuel et al., 2014; Grabowski & Moncrieff, 2002; Stephens et al., 2008; Tompkins & Craig, 1998a; Wing & Emanuel, 2014), while moderate vertical wind shear is detrimental to clustering (Held et al., 1993; Tompkins, 2001b). This research occurred in tandem with analyses of these feedbacks in other manifestations of convective organization and intraseasonal variabilities, such as the Madden‐Julian Oscillation or the Hadley/Walker cells (Bony & Emanuel, 2005). Wing and Emanuel (2014) introduced new diagnostics to quantify the relative role of these diagnostic feedbacks, and demonstrated how their relative importance could change during the phase prior to clustering onset and the mature organized state (see also Holloway & Woolnough, 2016; Muller & Bony, 2015; Wing et al., 2017). The occurrence or characteristics of convection clustering can be fickle; sensitive to domain size, resolution, and choices of subgrid‐parameterization approaches (Arnold & Putman, 2018; Jeevanjee & Romps, 2013; Muller & Bony, 2015; Muller & Held, 2012; Patrizio & Randall, 2019; Tompkins & Semie, 2017).

One motivation for the increase in interest in the spontaneous clustering of convection lies with its potential to alter climate feedback. For a given fixed surface temperature lower boundary condition, clustered states are much drier on average relative to one in which convection is randomly organized as the mean distance to convection increases (Bretherton et al., 2005; Held et al., 1993). Outgoing longwave radiation (OLR) thus increases with convective clustering. If clustering were to increase with mean surface temperature, this could represent a negative feedback on climate (Emanuel et al., 2014; Mauritsen & Stevens, 2015). This could be partly resolved if aggregation can be represented in global climate models (Bony et al., 2016; Coppin & Bony, 2015; Drotos et al., 2020; K. A. Reed et al., 2015), but potentially underestimated due to the inability for classical convective parameterizations to represent this on the subgrid‐scales. If the degree of clustering were to reduce with increasing mean temperature, this would instead represent a positive feedback on climate.

The climate feedback contribution of clustering is still an open question, since the relationship between clustering and the lower boundary condition is still poorly understood. Some studies have suggested clustering may strongly increase with lower boundary temperature (Wing & Emanuel, 2014). Emanuel et al. (2014) used an idealized model to suggest this could be a threshold process, with instability from radiative feedbacks with water vapor leading to clustering over warmer SSTs. In contrast, Wing and Cronin (2016) and D. Yang (2017, 2018) showed organization robustly occurring with SSTs ranging from 280 to 310 K, with the clustering scale reducing as SST increased, which would result in clustering acting as a *positive* climate feedback if atmospheric humidity increased with decreasing organizational scales.

Using fixed sea surface temperatures in the above idealized experimental frameworks prohibits spatial feedback of radiation with the spatially heterogeneous evolution of surface temperature, which could act to prevent, slow down or even amplify convective clustering, through shortwave anvil shading effects (Ramanathan et al., 1989), longwave interactions with the water vapor field, or latent and sensible heat anomalies that can impact the boundary moist static energy gradients and drive circulations in the planetary boundary layer (Hohenegger & Stevens, 2016; Shamekh et al., 2020; D. Yang, 2018). Some previous studies have examined this by including a slab ocean as a lower boundary condition. For example, Bretherton et al. (2005) conducted additional experiments with a slab ocean and reported that onset of clustering was delayed but not prevented with the slab ocean allowing spatial heterogeneity of SSTs. More recently, Hohenegger and Stevens (2016) came to a similar conclusion, as did K. A. Reed et al. (2015) using a global model. Recent simulations by D. Yang (2019) and Shamekh et al. (2020) emphasized the role of boundary layer processes in the delay caused by an interactive slab ocean. Y.‐T. Chen and Wu (2019) investigated aggregation in a model coupled to a slab ocean and showed how feedbacks could maintain strong SST gradients present in the initial conditions, while weak initial gradients would be removed by randomly located convection.

If a thin slab ocean is imposed, SSTs can vary significantly in response to solar diurnal forcing in clear sky conditions, which may then impact the convective diurnal cycle. In general, deep convection over the tropical oceans has an early morning maximum (Hall & Vonder Haar, 1999). Gray and Jacobson (1977) suggested spatial heterogeneities in longwave radiative heating between clear and cloudy regions may drive strongly convergence into the convective regions at night. A recent modeling study by Ruppert and Hohenegger (2018) provided more details on how both the heterogeneity in radiative heating and also the impact of cloud top cooling and SW absorption by water vapor on the vertical lapse rate may lead to the early morning peak in convection over maritime regions with thick ocean mixed layers (they imposed a fixed SST in their experiments to preclude surface feedbacks). Although there are numerous modeling studies that have been conducted to further our understanding of observations of the diurnal cycle of convection over land and ocean (Guichard et al., 2004; Ruppert, 2016; Ruppert & Johnson, 2016; Schlemmer et al., 2012; Stirling & Stratton, 2012; Vial et al., 2019) and Ruppert and Hohenegger (2018) investigated the diurnal cycle in both aggregated and non‐aggregated states, the possible role of the diurnal cycle in the onset of clustering has received less attention. Hohenegger and Stevens (2016) suggested that the diurnal cycle could delay the onset of clustering.

There is one aspect of the general experiment set up previously used to investigate interactive SSTs and the diurnal cycle that could be causing experimental outcomes to be misinterpreted and it pertains to the way the SST drift is constrained. In the tropics, there is a net export of heat by the ocean, which if neglected would lead to substantial drift in mean ocean temperatures with an interactive slab lower boundary. Although the studies of Bretherton et al. (2005), Hohenegger and Stevens (2016) and D. Yang (2019) applied a mean cooling through the application of a fixed Q‐flux to the slab ocean to prevent the mean temperature drifting excessively from the initial conditions, SST drift can not be completely constrained by this method.

If SST is free to evolve and the slab depth reasonably thick, the timescale to reach full equilibrium is long (T. W. Cronin & Emanuel, 2013), while for thin mixed layers the drift can occur on timescale of hours. This drift is important, as the occurrence and scale of convection self‐aggregation can potentially be impacted by small changes in the mean SST, and thus this experimental framework makes it difficult to isolate spatial‐surface feedbacks. Changes in the timing or nature of convective clustering attributed to the imposition of a slab ocean could be in part due to an ensuing change in the mean lower boundary temperature. Attempting to address this short‐coming, Shamekh et al. (2020) followed the approach of Semie (2016) in applying a fast (order minutes) timescale relaxation term to successfully constrain all SST drift. Unfortunately, this fast timescale also prohibits the thin slab ocean layers from responding to diurnal solar forcing. Thus if experiments do not apply a constant solar declination angle, they are effectively imposing an artificial diurnally varying energy flux in/out of the simulation domain, which is large in the case of a thin slab ocean. The objective of this paper is therefore to introduce a new experimental framework consisting of an adaptive Q‐flux method which completely prevents any drift in mean temperatures while at the same time allowing the surface to fully respond to diurnal forcing if desired. Using this new framework, we aim to investigate the role of an interactive ocean in the onset of convective clustering, both with and without diurnal forcing.

## 2 Model and Methods

### 2.1 Experiment Setup

The model used for the simulations is the non‐hydrostatic WRF model version 3.5.1 (Skamarock et al., 2008). The choices of parameterization schemes and experimental setup are the same as that used in Tompkins and Semie (2017), and are detailed in Text S1. To summarize the key points, a domain of 512 by 512 km is used with a horizontal resolution of 2 km, 62 vertical layers, and periodic lateral boundary conditions. The diurnal cycle is included, with the solar declination calculated assuming a nominal location of 0° longitude and 10° latitude.

The key difference to Tompkins and Semie (2017) is that the lower boundary consists of a slab ocean model. This is simple, modeling only a vertically uniform temperature with a fixed mixed layer depth (MLD) *h*. Density variations within the slab are neglected, and the model ignores the fresh water lenses that can result from intense convective precipitation and which can considerably alter surface heat exchange and mixing within the layer (Bellenger et al., 2017; Soloviev et al., 2015; Tomczak, 1995). The sea surface temperature SST is affected by the latent and sensible heat fluxes, in addition to the radiative fluxes, with the rate of change of ocean temperature given by:

(1) $$\frac{dSST}{dt}=\frac{F}{\rho_{l}C_{pl}h}+\frac{SST_{t}-\bar{SST}}{\tau },$$

where *F* is the net surface flux (positive downwards), consisting of the net latent and sensible heat and the LW and SW flux components, and *ρ*_*l*_ and *C*_*pl*_ are the density and specific heat capacity of water. Any imbalance in these four surface fluxes (non zero *F*) would lead to SST adjustment until equilibrium is achieved, which will be warmer than a typical warm pool surface temperature due to the neglect of lateral ocean heat transport as well as mixing through the thermocline. To represent this heat transport, the second term relaxes the domain‐mean SST (denoted $\bar{SST}$) toward a target value SST_*t*_ over a specified timescale *τ*. We emphasize that this term is spatially homogeneous and can only impact the domain mean SST, not spatial heterogeneity.

In this paper, we design two streams of experiments. In the first, we investigate the impact of spatial SST heterogeneities on clustering for a fixed boundary temperature of 28°C and a range of MLDs. In the second, we wish to additionally allow the SST to undergo a mean diurnal cycle, while still ensuring the *long‐term, multi‐day* mean SST is precisely maintained at the desired boundary temperature.

If *F* were time‐invariant, after an initial adjustment, $\bar{SST}$ would reach an equilibrium where $\frac{dSST}{dt}=0$. However, $\bar{SST}$ would not equal *SST*_*t*_. Instead, from Equation 1 it is apparent that the equilibrium $\bar{SST}$ would be offset from SST_*t*_, with the magnitude of the bias given by $\frac{\tau F}{\rho_{l}C_{pl}h}$. If *τ* is small (∼ minutes), this offset, or “drift”, is limited and $\bar{SST}$ will remain close to SST_*t*_. However, if *τ* is set to a value ∼ *O*(1 day) to allow diurnal variations in $\bar{SST}$, the difference between the long‐term (>1 day) mean SST and SST_*t*_ can be appreciable (refer Figure S1), depending on magnitude of the forcing *F* and MLD *h*.

It can not be overemphasized how critical it is to strictly prevent any SST drift in the experimental framework. Previous studies have shown how the mean SST can impact the occurrence or scale of convective clustering (e.g., Wing & Cronin, 2016) and preliminary investigations with a range of fixed lower boundary temperatures showed that clustering in the WRF is also highly sensitive to the lower boundary temperature. Thus avoiding drift in mean SST is crucial to avoid it confounding the signal of spatial anomalies and the mean diurnal cycle.

If we use the notation SST_*b*_ to represent the desired boundary temperature of 28°C, the desired boundary temperature could in theory be achieved by specifying a value for SST_*t*_ that is colder than the desired boundary condition SST_*b*_:

(2) $$SST_{t}=SST_{b}-\frac{\tau F}{\rho_{l}C_{pl}h}.$$

Bretherton et al. (2005) essentially used this approach to reduce drift by applying a fixed energy sink to the slab ocean. In practice, as *F* changes in time, undergoing a diurnal cycle and also evolving in tandem with the model mean state, it is problematic to calculate the offset required to eliminate all SST drift. We therefore introduce a new approach of an adaptive system by making SST_*t*_ itself prognostic, evolving to slave the long term running mean $\bar{\bar{SST}}$ toward SST_*b*_ according to:

(3) $$\frac{dSST_{t}}{dt}=\frac{SST_{b}-\bar{\bar{SST}}}{\tau_{adj}}.$$

$\bar{\bar{SST}}$ is an average both over space, but also over a time scale that exceeds the diurnal cycle. We calculate $\bar{\bar{SST}}$ as an exponential moving average using Welford's algorithm with a window of 12 h *τ*_*adj*_ should exceed the averaging timescale of $\bar{\bar{SST}}$ for stability reasons and is set to 1 day. These values were found to be optimal by ensuring that diurnal variations are not reduced in magnitude by more than a few percent while at the same time minimizing the initial adjustment time, with $\bar{\bar{SST}}$ adjusting to SST_*b*_ within the first 2 days of integration. To minimize ocean spin up, we initialize the SST to SST_*b*_, while the initial value for SST_*t*_ is based on Equation 2 using an estimate of the surface flux imbalance. Further information on this framework is given in supplementary information Figure S1. We note that this method could be used with a longer timescale than 12 h to allow modes of ocean variability on bi‐diurnal timescale (Yu et al., 2018) or even longer multiday timescale associated with mesoscale systems if desired, while still eliminating all drift on longer timescales.

**Figure 1:**
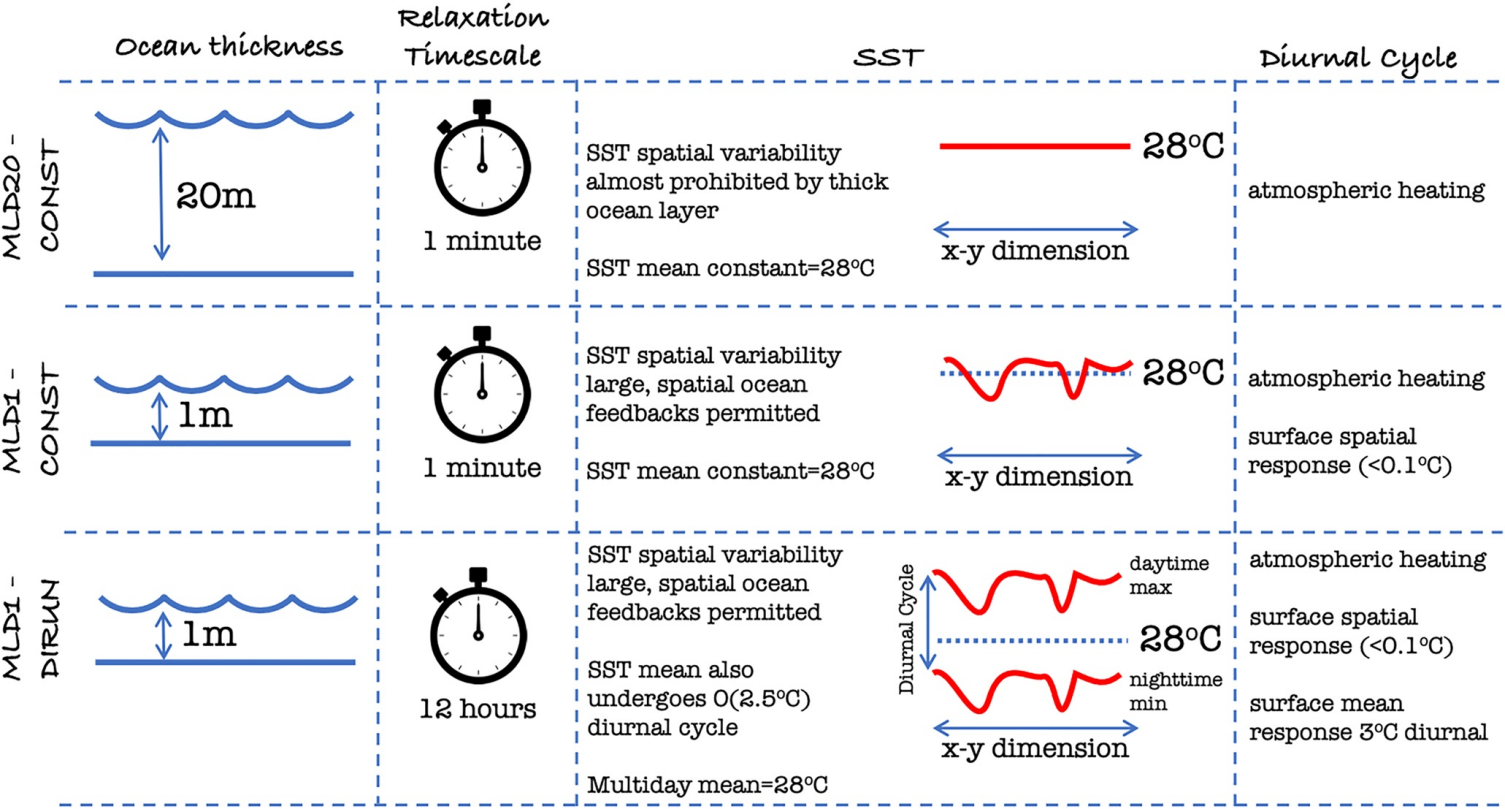
Configuration of the three key experiments conducted. Each of these experiments consists of an ensemble of six simulations to evaluate the variation in clustering onset times. In addition, a set of single integrations were conducted with intermediate mixed layer depths of 5, 10, and 20 m and a 1 min relaxation timescale that lie between MLD20‐CONST.

The key experiments conducted in this work are summarized schematically in Figure 1. In the first experiment we set *τ* = 1 min; a fast adjustment that prohibits a diurnal cycle in the *domain‐mean* SST, which remains constant, and in fact, for this short timescale the above system essentially reduces to a simple Newtonian relaxation, as SST_*t*_ ≈ SST_*b*_. The 100 days simulations conducted for this experiment are referred to as MLD*h*‐CONST, with *h* indicating the depth of the ocean mixed layer employed in each experiment. Two main experiments use 20 and 1 m ocean MLDs, each including an ensemble of six simulations to test for stochastic variability in clustering onset, and a further 5 days extension was made to one member including additional diagnostics of the clustered state. In addition to these three key ensemble experiments, three single supplementary experiments are conducted that are not included in Figure 1 for reasons of brevity. The first applies a constant SST lower boundary, equivalent to an infinite MLD, while the second and third use intermediate MLDs of 10 and 5 m, respectively. We should emphasize that in these experiments, the atmosphere still undergoes a diurnal cycle of radiative heating and cooling, and the surface undergoes a diurnal cycle in the *spatial* radiative forcing; it is only the *domain mean* SST diurnal variation that is prevented.

In the second experiment, we perform an additional ensemble of six simulations setting *τ* = 12 h, which permits a diurnal cycle in the domain mean SST *in addition* to the spatial feedbacks already represented in the MLD*h*‐CONST experiments, while the multi‐day mean SST ($\bar{\bar{SST}}$) does not depart more than 0.01 K from SST_*b*_ after 2 days (see Figure S1). The impact of the diurnal heating on the ocean surface temperature would be minimal in cases where disturbed conditions with higher winds drive a deep mixed layer (Bernie et al., 2005; Sui et al., 1997), hence the mean diurnal cycle experiments are only conducted for the thinnest mixed layer of 1 m, referred to as MLD1‐DIURN.

In summary, the MLD20‐CONST experiments prevent almost all surface feedbacks due to the unresponsive mixed layer, MLD1‐CONST then adds spatial surface feedbacks, and finally, MLD1‐DIURN allows both spatial and mean SST feedback's.

### 2.2 Diagnostics

#### 2.2.1 Column Classification

We use a system of column classification to simplify the analysis by dividing the troposphere into the lower (surface to 600 hPa) and upper (600–100 hPa). These layers are classified as moist or dry using a slab column integrated relative humidity (*∫q*_*v*_/*∫q*_*s*_) of 60% and cloudy/clear using a total column integrated cloud liquid and ice water threshold of 1 g m^−2^. From the 16 possible states, only six have significant occupancy referred to as deep moist convection, low cloud moist, high anvil, moist clear, dry upper clear, and dry column clear, which are detailed in Table 1.

**Table 1:** Categories of Columns

Name	Humidity		Cloud	
	Lower	Upper	Lower	Upper
Deep convection	Moist	Moist	•	•
Anvil high cloud	Moist	Moist	○	•
Low cloud	Moist	Moist	•	○
Clear moist	Moist	Moist	○	○
Clear dry upper	Moist	Dry	○	○
Clear dry	Dry	Dry	○	○

*Note*. Lower/Upper refers to the lower troposphere (surface to 600 hPa) and upper troposphere (600–100 hPa) while •/○ refer to cloudy or clear conditions, respectively.

#### 2.2.2 Convective Organization

Several metrics of convective organization have been reviewed by Holloway et al. (2017). We utilize the following metrics of convection and its organization, each with its specific advantages:

- Domain mean OLR: This highlights how the onset of clustering impacts the energy budget, and is a reliable indicator of the onset timing.
- Spatial variance of SST: The heat capacity of the ocean slab means this index is also a smooth indicator of when onset occurs compared to (noisier) metrics based on convective velocity or cloud properties, but is subject to a variable lag relative to OLR or velocity based metrics.
- *I*_*org*_: Introduced in Tompkins and Semie (2017), calculated from the cumulative nearest neighbor distances between all updraft cores (columns with *w*_700 hPa_ > 1 m s^−1^), this index is an absolute metric of organization, taking the value 0.5 for randomly distributed convection and values exceeding (less than) this for clustered (regular) convection.
- *d*_95_: The distance to the nearest convective column (a column with updraft velocity exceeding 1 m s^−1^ at 700 hPa) is calculated for all columns in the domain. This metric is the 95th percentile of the resulting distribution, that is, 95% of columns are within this distance to a convective moistening source, and is a measure of the ubiquity of local convective moistening sources.

#### 2.2.3 Timing of Onset

For robustness, the time of onset of clustering (TOC) is calculated using four different methods, the first two based on the domain‐mean, daily mean OLR time series, the third on the humidity distribution, and the last using the vertical velocity field.

- OLR 3*σ*: Calculates the linear trend fitted using the first 30 days (or 15 in the case of MLD20‐CONST) and the standard deviation *σ* of the detrended daily mean OLR series. The onset is then the first model step that the OLR departure from the extrapolated linear trend exceeds +3*σ*.
- Walker‐Bordoni Index (WBI): Applies the first order change‐point analysis of Walker and Bordoni (2016) on the initial part of the series where OLR < 262 W m^−2^ and is referred to as the WBI.
- Dry%: This method uses the column decomposition to assess humidity variance, and defines onset as the first time in which the domain coverage of the clear dry columns category exceeds 1%.
- *I*_*org*_: We examine a moving 5 days window of *I*_*org*_ values (20 scenes separated by 6 h) and then apply a one‐sided Student's *T* test to judge if the mean *I*_*org*_ value is greater than 0.5 which would imply an exactly random field. The onset is defined as the center of the first 5 days window where the null hypothesis of random convection is rejected at the 99.9% significance level. Implicit in this is the assumption that fields separated by 6 h are independent random samples, which is reasonable in the pre‐onset state when the temporal autocorrelation of humidity perturbations is short. The possible overestimation of the degrees of freedom due to this assumption is compensated for by selecting a very high significance level to ensure statistical robustness. We note that we could have alternatively used the classical Clark‐Evans statistical test (Clark & Evans, 1954) directly on sub‐sampled nearest neighbor distances of the velocity field, however, we prefer the application to *I*_*org*_ as it is a scene‐integrated quantity that has been widely applied in the field since its introduction (e.g., Bony et al., 2020; Brueck et al., 2020; Janssens et al., 2021; Moseley et al., 2020; Pscheidt et al., 2019).

#### 2.2.4 Surface Flux Decomposition

Spatial variability in surface latent heat fluxes can derive from changes in surface wind speed measured at the 10 m height, *V*_10_, and the degree of the humidity mixing ratio deficit with respect to the ocean saturated value Δ*q* = *q*_*sat*_(SST) − *q*_2_, where 2 and 10 refer to the measurement height (although many models use the value from the lowest available model level in flux parameterizations).

In models that employ a simple bulk aerodynamic formula for the latent heat flux *F*_*lh*_(*v*, Δ*q*) = *L*_*v*_ *ρc*_*d*_ *V*_10_Δ*q* (where *c*_*d*_ is the drag coefficient), it is straightforward to decompose the relative contributions of velocity and flux perturbations $F_{lh}\left(V_{10}^{\prime }\Delta \bar{q}\right)$, $F_{lh}\left(\bar{V_{10}}\Delta q^{\prime }\right)$ and the correlation residual term *F*_*lh*_(*V*_10_′Δ*q*′), where the overbar and ′ represent the domain mean and perturbation quantities, respectively. Instead, WRF employs a more advanced approach based on similarity theory, which accounts for the Richardson number changes through empirical stability functions. To decompose the fluxes into the contribution from local perturbations to the wind and boundary layer thermodynamic properties, rather than attempt to drive the full complex WRF scheme in offline mode, we simplified the procedure by instead using a machine learning approach to approximate the WRF scheme, following earlier work by Gentine et al. (2018); Krasnopolsky et al. (2005) and Rasp et al. (2018). Using the MLPRegressor function of the sklearn package (release 0.23.2) of python 3.6.9, we trained a neural network with 4 hidden layers of 64 neurons. The network was trained using 80% of the data from 4 time slices taken from the early, middle, and late stages of the MLD1 simulation (0.8 × 2^18^ data points), with the remaining 20% used for independent validation of the neural network. The validation revealed a *r* ^2^ correlation of 0.999, a RMSE of 0.2 W m^−2^ and a mean bias of −0.05 W m^−2^ and is illustrated in Figure S2. The impact of perturbations for each wind, Δ*q* and Δ*T* is calculated by using the value of the variable measured in each location in combination with the domain‐mean value of the other two variables to ensure the neural network is only used for values within the range of the training data.

## 3 Convective Aggregation

**Figure 2:**
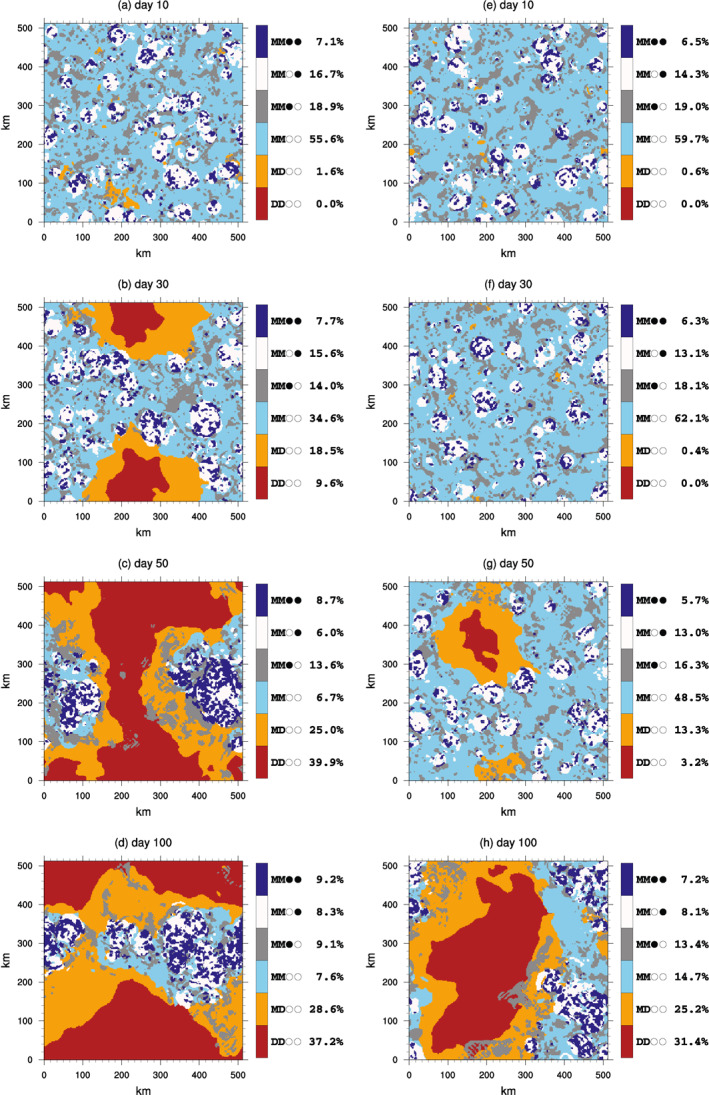
Slices showing domain snapshot at (a) 10, (b) 30, (c) 50, and (d) 100 days of member 0 of the MLD20‐CONST experiment, with panels (e–h) showing the same periods for member 0 of MLD1‐CONST, with each column classified as one of six categories of deep convection, high anvils, shallow cloud, clear moist, clear dry upper, and clear dry.

**Figure 3:**
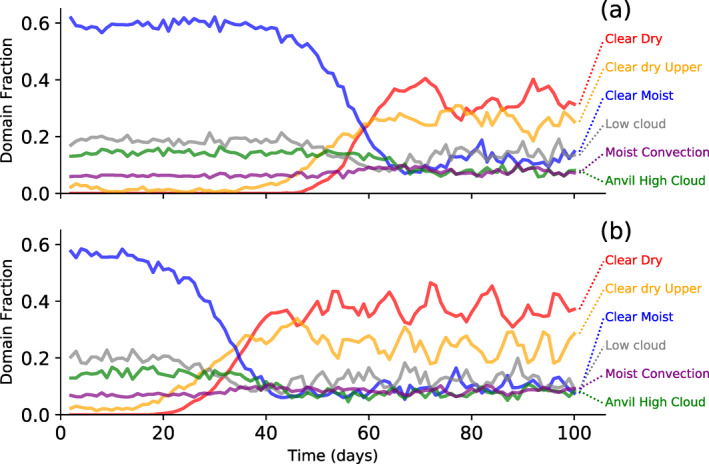
Time series showing evolution of the six column classifications in member 0 of the (a) MLD1‐CONST and (b) MLD20‐CONST experiments. See text for details of column definition.

We first analyze the MLD20‐CONST experiment to quantify how aggregation occurs and the role of diabatic feedbacks. Maps of the six columns states of clear/cloudy and moist/dry show the evolution of the deep and shallow convection locations in member 0 of the MLD20‐CONST (Figure 2, left column) and of MLD1‐CONST (right). On day 10, prior to clustering onset, the deep moist cloudy columns cover around 7% of the domain in both experiments. This is higher than the fraction of the domain that is, covered by dynamical updrafts as defined by columns with 700 hPa updraft velocities exceeding 1 m s^−1^, which will be shown later to be on the order of 0.4% (in line with the scale analysis of Craig, 1996; Tompkins & Craig, 1998a), since the cloud fractional area includes dynamically inactive cloudy columns as well as downdrafts. The deep moist columns are surrounded by upper anvil cloud covering approximately 15% of the domain, while scattered low cloud covers an additional 20% of the simulation domain. As the convection starts to organize, a dry upper clear patch forms and grows with time, deepening and eventually also impacting the lower troposphere, and eventually the convection is restricted to a limited region of the domain (as in Bretherton et al., 2005; Muller & Held, 2012; Tompkins & Craig, 1998a), surrounded by a transition zone of moist lower, dry upper clear columns which separate the convective area from the dry regions. The temporal evolution of the six classes reveals the clear moist classification occupying approximately 60% of the domain at the start of the simulation when convection is random, referred to as the “pre‐onset phase” hereafter (Figure 3). Prior to day 20, the fraction of moist‐dry clear starts to increase, followed shortly after by the dry‐dry clear columns, which is used in one of the definitions of clustering onset. We note that the exact onset time will depend to some extent on the thresholds used to define these categories.

**Figure 4:**
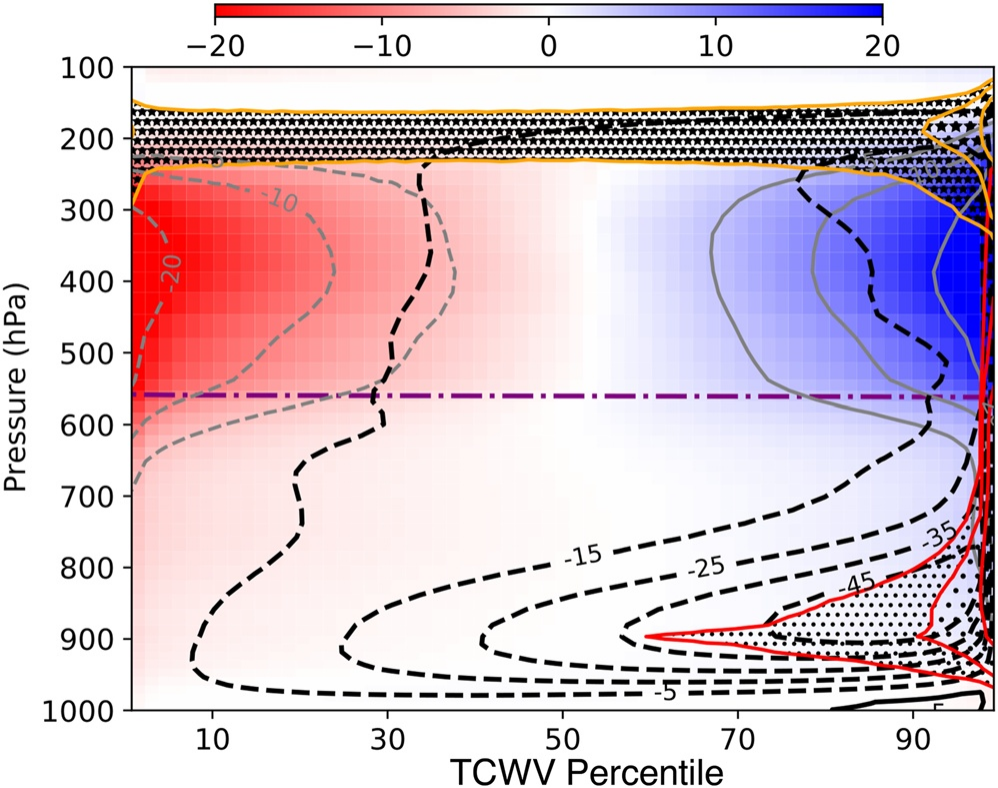
MLD20‐CONST Day 3–10 average of normalized water vapor perturbation (color shading and gray contours), liquid water mixing ratio (dot shading and red contours marking 10^−5^, 3.10^−4^, and 10^−4^ kg kg^−1^) and ice water mixing ratio (star shading and orange contours marking 10^−6^, 3.10^−5^, and 10^−5^ kg kg^−1^) as a function of the TCWV percentile. Black contours show the mass flux streamlines as defined by Bretherton et al. (2005) (units 10^−2^ kg m^−2^ s^−1^).

In the pre‐onset phase, the largest normalized perturbations in humidity appear in the upper half of the troposphere above the freezing level above 600 hPa in a mode two like pattern (Figure 4). An examination of a time‐height evolution of domain means humidity shows that this upper tropospheric perturbation appears approximately contemporaneously at all heights between 600 hPa and the detrainment level (not shown). The streamlines show a shallow circulation associated with shallow convection located in the moistest part of the domain as well as the circulation associated with deep convection to the troposphere. While the largest relative anomalies in column humidity are in the upper troposphere, the variation in lower tropospheric humidity is sufficient to give a range of total column water vapor (TCWV) between approximately 50 and 70 kg m^−2^.

**Figure 5:**
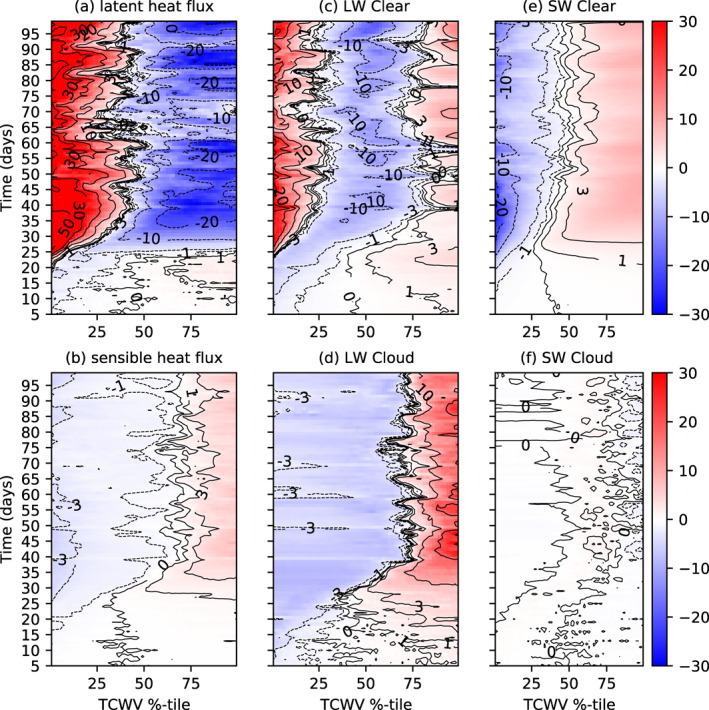
Hovmöller diagram of atmospheric convergence spatial anomaly (positive is warming anomaly of atmosphere) for MLD20‐CONST. (a) Surface latent heat, (b) surface sensible heat flux, and net atmospheric convergence of (c) LW clear sky flux, (d) LW cloud forcing (total flux ‐ clear), (e) SW clear, and (f) SW cloud fluxes of member 0 of the experiment MLD20‐CONST, ordered according to TCWV sampled four times a day.

Examining the spatial perturbation of the atmospheric heating, sorted by TCWV, can reveal the nature of the diabatic feedbacks that are forcing or opposing clustering, similar to the approach of Wing and Emanuel (2014). The diabatic terms consist of the surface latent and sensible heat fluxes and the LW and SW net atmospheric radiative flux convergence (top of atmosphere (TOA) minus surface) divided in their respective clear sky and cloud forcing contributions (Figure 5). Heating perturbations in the moist columns would indicate a process driving convergence and encouraging aggregation. In agreement with the analysis of Wing and Emanuel (2014), several processes contribute to aggregation. Both the latent and sensible heat fluxes are slightly enhanced in the moist areas in the pre‐onset phase (Figures 5a and 5b), which suggests that they weakly contribute to clustering. However, once clustering commences and the dry anomaly develops, the role of latent heat fluxes reverses and acts to oppose clustering, as also noted in Wing and Emanuel (2014). Instead, the warm boundary layer and cold SSTs under the dry patch suppress the sensible heat flux there, and thus enhance the sensible heat flux contribution to clustering in the organized state.

The clear‐sky longwave flux convergence (Figure 5c) also agrees with the findings of Wing and Emanuel (2014), Emanuel et al. (2014), with clear‐sky, longwave cooling enhanced in dry areas in the early stages of clustering onset (day 0–20) due to the drying upper troposphere. Once the drying impacts the lower troposphere, significantly reducing lower tropospheric emissivity, a relative warming commences there (Figure 5c), while the moistest regions also are subject to a warming anomaly. The maximum in the cooling rate occurs over the intermediate values of water vapor. In tandem, the LW cloud forcing also drives convergence into the moist regions (Figure 5d) in the early stages of the simulation, with the cloud forcing strengthening as aggregation begins to establish. In the aggregated state this represents the strongest diabatic term for maintaining organization, as documented by Stephens et al. (2008). The SW clear‐sky heating anomalies also act to promote clustering in the equilibrium state due to enhanced SW absorption in the moistest region, with anomalies of 15–20 W m^−2^ across the domain in the clustered state. Instead, the reduction in SW column heating due to cloud forcing (high clouds reducing incoming solar), opposes clustering but the effect is very weak. The fact that both surface fluxes and LW clear/cloud and SW clear sky appear to drive clustering would also appear to be consistent with the early experiments of Tompkins and Craig (1998a), who found that preventing spatial heterogeneity in either surface fluxes or radiative heating prevented aggregation, with radiative forcing the more dominant of the two.

**Figure 6:**
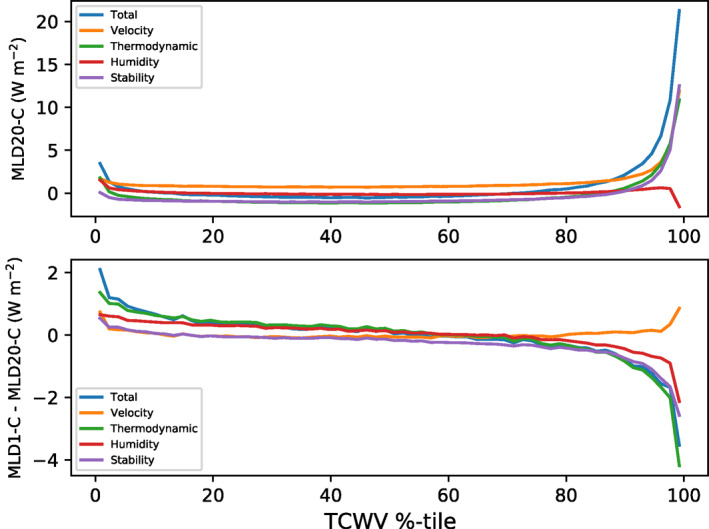
Contributions to the latent heat surface flux perturbations during day 3–10 in the pre‐onset phase of (a) MLD20‐CONST and (b) MLD1‐CONST minus MLD20‐CONST differences, from wind perturbations, humidity (Δ*q*) and stability (Δ*T*) differences, where Δ*q* is the difference between the saturated mixing ratio at the sea surface and the mixing ratio of the lowest model level, and Δ*T* is calculated as the difference between the SST and the two meter temperature. (CONST is abbreviated to "C" in the axis labels). Thermodynamic perturbations refer to the contribution from Δ*T*, Δ*q*, and the Δ*T*Δ*q* terms. Calculations are made using a neural network fitted to the WRF full flux calculations (see methods).

To understand the surface latent heat flux anomalies in the pre‐onset phase, we now decompose them into the contribution from boundary layer wind speed, moisture gradients between the ocean surface and boundary layer, and the low level stability using the neural network (Figure 6a, see methods for details). In the MLD20‐CONST experiment, the boundary layer velocity perturbations only impact surface fluxes in the vicinity of deep convection, due to enhanced gustiness associated with cold pools (orange line). Boundary layer humidity has a limited impact on the surface fluxes in the pre‐onset state, probably due to the fact that the cold pool structure consists of both dry anomalies surrounded by enhanced moisture rings, first noted in simulations by Tompkins (2001a), which would have a compensating effect, as discussed in Hirt et al. (2020) and Torri et al. (2015). In contrast, cold pools only have a negative signature on boundary layer buoyancy. Cold pools increase the temperature difference between the SST and the boundary layer temperature, leading to a stability‐enhanced latent heat flux that is, similar in magnitude to the velocity contribution. We note that this impact on the surface fluxes would be absent in a model using a bulk aerodynamic formula that ignores stability.

In the clustered state, the forcing due to latent heat fluxes was seen to reverse with latent heating opposing the organization (Figure 5a). Applying the neural network flux model to the clustered state shows that this reversal is completely dominated by the boundary layer water vapor structures, compared to which the stability and wind components are negligible (see Figure S3a). The strong subsidence in the cold SST area impacts also the humidity of the boundary layer and to such an extent, that increases the latent heat flux despite the depressed SSTs.

In summary, the results of the experiments over thick mixed layers where SST spatial variability is highly constrained confirm the findings of previous investigations in terms of the structure of the aggregation and the role of surface fluxes and radiative forcing (particularly LW forcing from water vapor and clouds) in driving aggregation of convection. The neural network disassociation of the surface flux effects shows that a WISHE‐like wind‐driven feedback is important, but that lower level stability also plays a role that would be absent in models that use simple bulk flux schemes.

## 4 Impact of Ocean Feedbacks

In this section, we examine the impact of reducing the ocean thickness to 10, 5, and then finally the thinnest layer of 1 m (the MLD1‐CONST experiment with six ensemble members). Maps of the column classification in the 1 m case show that convection still undergoes aggregation, even in the thinnest mixed layer case, but that the clustering appears to take longer to establish, even though the nature of the clustering is similar (Figure 2, right column). Prior to clustering onset, the fractional coverage of each classification is very similar between MLD20‐CONST and MLD1‐CONST (Figure 3b), but the delay in the onset of clustering is longer in the experiment using a thinner MLD, with the increase in the Clear‐Dry Upper and Clear Dry categories occurring around day 40. The low cloud fraction appears to be marginally higher in the pre‐onset, early period of the MLD20‐CONST experiment, which could have an impact on organization given the emphasis placed on the role of low cloud radiative forcing in Coppin and Bony (2015) and Muller and Bony (2015). While a thinner ocean mixed layer delays the point of organizational onset, it does not appear to significantly change the timescale of the dry patch growth in the initial transition phase. This is not inconsistent with the idealized, coarsening model of Windmiller and Craig (2019) that suggests that the growth rate is controlled by diffusive processes, at least in the early stages of clustering onset.

**Figure 7:**
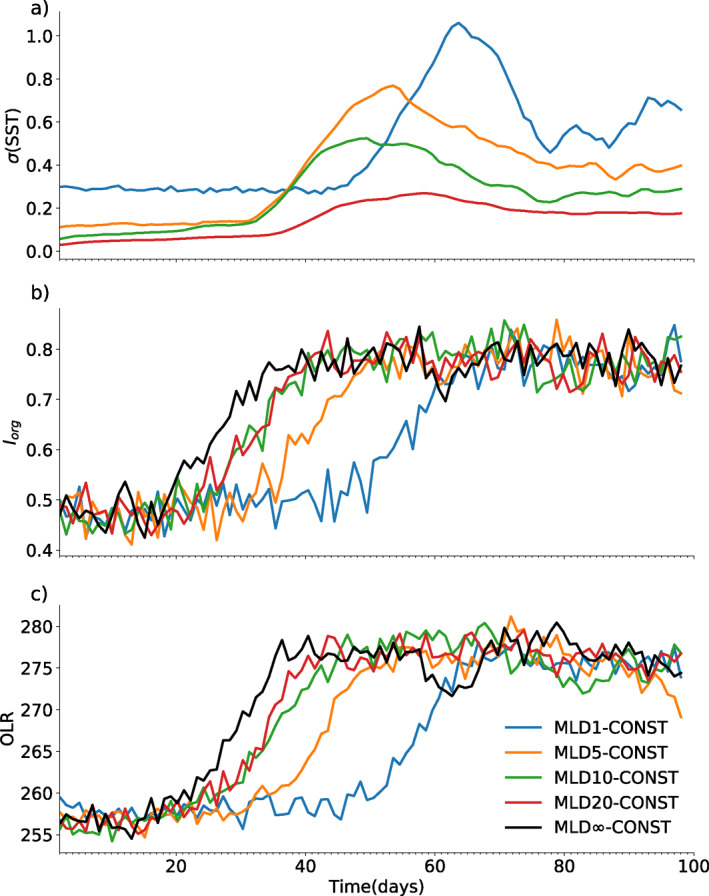
Time series of daily (a) spatial standard deviation of SST (b) degree of the organization measured by *I*_*org*_ (c) domain mean outgoing longwave radiation (OLR), for member 0 of each MLD*X*‐CONST experiment (see legend).

The metrics of *σ*(SST), *I*_*org*_ and $\bar{OLR}$ are shown in Figure 7 for member 0 of all MLD*h*‐CONST experiments, showing the full range of ocean mixing layer depths. The spatial standard deviation in SST in the early period of the experiments reflects the behavior expected, with greater variability for thinner MLDs. As clustering onset begins, variance in SST increases, which will be shown to be due to temperatures decreasing under the forming dry patch where deep convection is suppressed. The *I*_*org*_ organization index clearly shows the systematic impact of a thinner mixed layer on delaying the onset of clustering (Figure 7b). According to the *I*_*org*_ measure for TOC, the mean onset over all six ensemble members is day 37.5 for a mixed layer of 1 m (MLD1‐CONST) compared to day 24.8 for a 20 m mixed layer (MLD20‐CONST) (Table 2). The TOA OLR flux (Figure 7c) also increases with the drying associated with clustering onset by approximately 20 W m^−2^ (Bretherton et al., 2005; Holloway et al., 2017; Wing et al., 2017). The comparison of the TOC roughly agrees across all four definitions, ranging from 21.7 to 27.5 days for the MLD20‐CONST, and a standard deviation of approximately 2–3 days estimated from the six ensemble members (Table 2). Instead for the MLD1‐CONST TOC ranges from 36.9 to 45.7 with a standard deviation of 4.9–6.4 days. Thus the delay in clustering onset is highly statistically significant.

**Table 2:** Clustering Onset Time and Standard Deviation as Calculated by Three Contrasting Methods: The First Two are Based on OLR, the Walker‐Bordoni Index (WBI) and the 3σ Method (3σ), the Third Method is Based of the Time the Fractional Coverage of Dry‐Dry Columns Exceeds 1% (Dry%), and the *I_org_* Method Chooses the First Time Slice When the Null Hypothesis That the 5 days Average *I_org_* Value (See Tompkins et al., 2017, for Calculation Method) Represents a Random Distribution is Rejected in Favor of Clustered Convection at the 99.9% Significance Level. (Refer to Methods Section for Details)

Experiment	Mean onset (days from simulation initialization)				Standard deviation of onset (days)			
	WBI	3*σ*	Dry%	*I_*org*_*	WBI	3*σ*	Dry%	*I_*org*_*
MLD20‐CONST	24.2	27.5	21.7	24.8	3.0	3.0	2.5	2.2
MLD1‐CONST	44.5	45.7	36.9	37.5	6.4	6.2	6.2	4.9
MLD1‐DIURN	43.0	44.2	37.5	40.4	9.5	9.5	10.3	9.4

**Figure 8:**
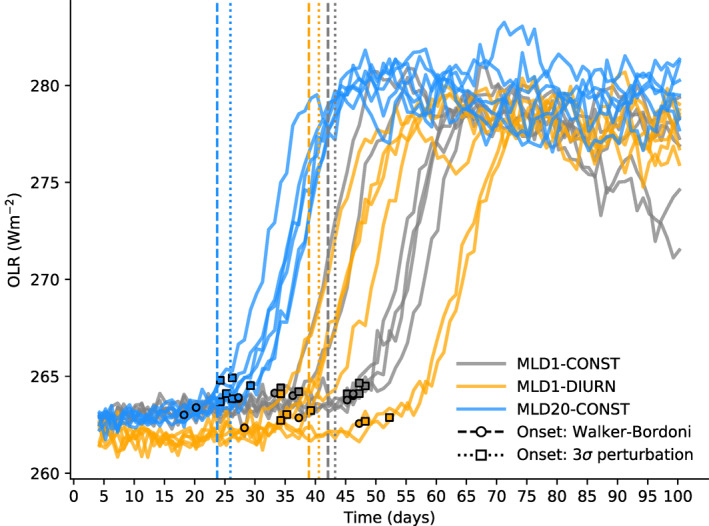
Daily mean OLR for the six ensemble members of simulations for MLD20‐CONST, MLD1‐CONST, and MLD1‐DIURN experiments. The points mark an estimated clustering onset time calculating the time when OLR exceeds the extrapolated linear trend of day 1–30 by more than three standard deviations (calculated from the detrended day 1–30 series). The average is marked with the dotted vertical lines. For comparison, the dashed lines show the average clustering onset time defined using the first order piece‐wise change‐point analysis described by Walker and Bordoni (2016) applied to the OLR series (cut at the point when it exceeds 262 W m^−2^).

The clustering onset in Figure 7 does not increase monotonically with ocean MLD, with the onset timing similar for MLD10‐CONST and MLD20‐CONST for example, indicating some degree of stochastic variation. This variation is revealed by examining the six‐member ensembles for the key experiments. While the ensembles are limited in size, they are sufficient to document a considerable variation in the onset timing of clustering for some experiments (Figure 8). When the clustering onset occurs at around day 25 as in the thicker mixed layer experiment MLD20‐CONST, there is little variation between the experiments (Table 2). Instead, when the use of a thin layer ocean delays the onset to around 40 days, the timing becomes much more variable, as seen in the MLD1‐CONST and also the MLD1‐DIURN experiments discussed next. This change in the variability of aggregation onset time is the expected behavior if we envisage the system as bi‐stable with hysteresis and is subject to noise associated with the convective triggering. Muller and Held (2012) and Y.‐T. Chen and Wu (2019) have previously demonstrated hysteresis and sensitivity to initial conditions in cloud resolving investigations of aggregation. In the MLD20‐CONST experiment, where the diabatic forcing for convection aggregation is stronger and the onset of clustering occurs in the minimum possible time set by the tropospheric subsidence mixing timescale (T. W. Cronin & Emanuel, 2013; Tompkins & Craig, 1998b), the impact of stochasticity is small. As shown by Ditlevsen and Johnsen (2010) with a double‐welled Langevin equation and constant forcing, the phase transition to clustering will occur earlier than the bifurcation point due to the noise but this advance will depend on the magnitude of the noise relative to the forcing. Thus as the ratio of the noise to the forcing increases the variability of the transition will increase until it is either eliminated (clustering does not occur) or becomes entirely random (as in Figure 2b of Ditlevsen & Johnsen, 2010). This is also shown clearly in Figure 5 of Thomas and Jones (2016) with weakly forced systems (or with large relative noise amplitude) undergoes a much higher degree of stochasticity in the transition time. Thus in the experiments with a thin mixed layer, which weakens the diabatic forcing for clustering the noise/forcing ratio is increased and the timing at which the transition to clustering occurs increases significantly from 2 to 3 days to around 5–10 days, depending on the experiment and metric for TOC. Further investigation of what constitutes the threshold for organization is required.

**Figure 9:**
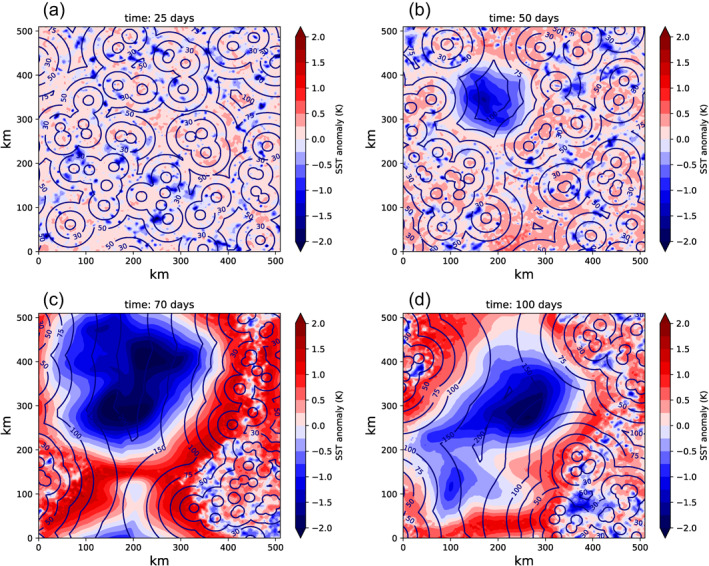
SST maps for days (a) 25, (b) 50, (c) 70, and (d) 100 days of member 0 of the MLD1‐CONST experiment. The contour lines show the distance to the nearest updraft core (units km) defined by a vertical velocity exceeding 1 m s^−1^ at 700 hPa.

Time‐slices of SST for the MLD1‐CONST experiment reveal the nature of the increases in SST‐variance as convective clustering occurs (Figure 9), with isolines showing the distance to the nearest convective updraft column. In the early stages of the experiment, prior to clustering onset (day 25, Figure 9a), SST anomalies are mostly less than 1 K in magnitude and are not spatially coherent. Small cold anomalies are associated with the action of cold pools, with the ocean able in this case to react on short timescales to the wind and stability driven enhancements of surface fluxes and cool rapidly. Once clustering onset has started (day 50, Figures 5b), a patch of cold sea surface temperatures forms under the region of dry atmosphere. By day 70 (Figure 5c) the dry patch cold anomaly exceeds 2 K, and an interesting structure in SSTs has been established. Dividing the deep convective locations (marked by cold pool structures) and the large cold SST patch, there is a border zone of large bands where the SST is hottest. By day 100, the patterns are similar, with evidence of strongly organized cold pools in the convective region (Figure 9d).

Cold pools are a critical component, that impact boundary humidity and buoyancy structures, cool the underlying sea surface and can potentially play an important role in delaying clustering onset (Gentine et al., 2016; Jeevanjee & Romps, 2013; Schlemmer & Hohenegger, 2014, 2016; Tompkins, 2001a; Torri et al., 2015). Windmiller and Hohenegger (2019) emphasized the role of these cold pools, collectively forming a so‐called super cold pool, and suggested that in their simulations, the suppression of convection in the super cold pool region by negative buoyancy anomalies lead to the enhanced convection being found at the boundaries of the moist zone, much the same mechanism as suggested by Nakajima and Matsuno (1988) in an early study of organization. The role of the interactive ocean used in their simulations was discounted by Windmiller and Hohenegger (2019), but the strong impact on surface fluxes in the organized state would also act to enhance convection on the moist region edges, as also seen in Shamekh et al. (2020). The development of zones of maximum SST between the convective area and the dry regions are emphasized in Hovmöller plots, which rank the SST as a function of the TCWV percentile (Figure S4). In the aggregated state, the convection remains in the areas of highest moisture and thus moist static energy, while the warmest SSTs are found in the adjacent regions which will be shown to be moist but clear sky. We also highlight the SST cold region, and the spatial SST variance reaches a maximum just after clustering onset in both experiments, before reducing after about 20–25 days, similar to the overturning time of the troposphere (Tompkins & Craig, 1998b).

**Figure 10:**
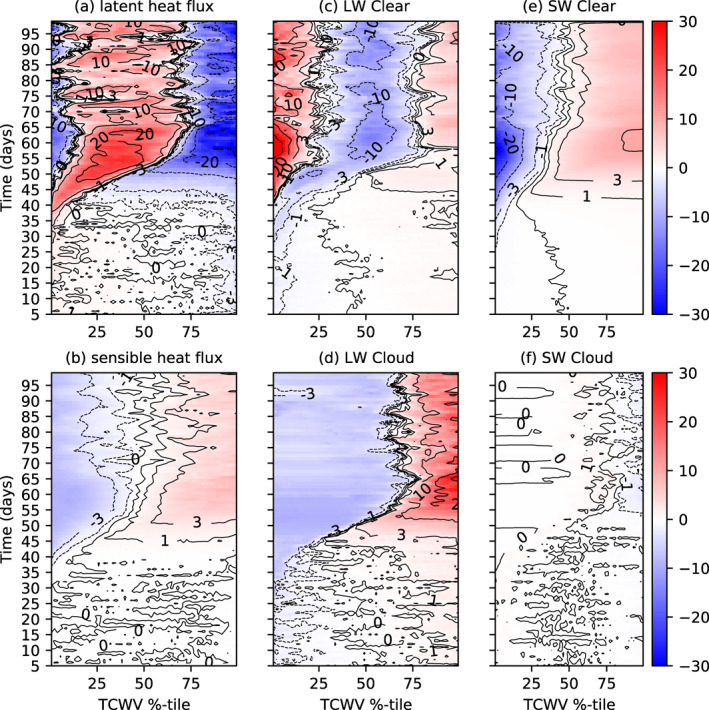
Hovmöller diagram of atmospheric convergence spatial anomaly (positive is warming anomaly of atmosphere) for MLD1‐CONST. (a) Surface latent heat, (b) surface sensible heat, and net atmospheric convergence of (c) LW clear sky flux, (d) LW cloud forcing (total flux ‐ clear), (e) SW clear, and (f) SW cloud fluxes of member 0 of the experiment MLD20‐CONST, ordered according to TCWV sampled four times a day.

With the thin reactive ocean layer of 1 m, the structure of the SW and LW atmospheric forcing is similar albeit weaker than the MLD20‐CONST control integration (Figures 10c–10f, compare to Figure 5), with the delayed onset of clustering apparent. However, the interactive surface temperature that results in the strong cooling under the developing dry area has a significant impact on the surface latent and sensible heat fluxes, resulting in negative latent heat flux anomalies over the driest regions in the clustered state. Another key difference is the role of surface fluxes in the pre‐clustered state, with the no gradient in sensible heat fluxes apparent prior to day 30 due to the fast adjustment of the surface, while the latent heat flux actually opposes clustering in the early stages of the experiment instead of favoring aggregation as in MLD20‐CONST.

**Figure 11:**
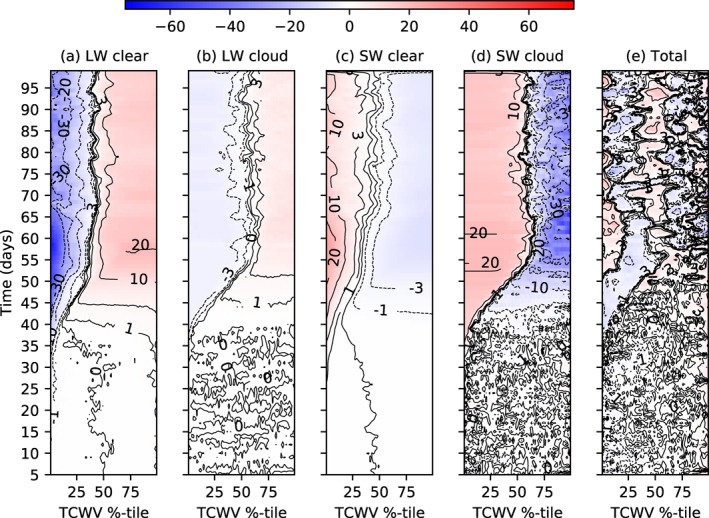
Surface fluxes anomaly (W m^−2^, positive = downward) in member 0 of experiment MLD1‐CONST of (a) Clear‐sky LW, (b) LW cloud forcing, (c) clear SW, (d) SW cloud forcing, and (e) total surface flux anomaly (SW + LW + LH + SH).

The surface flux anomalies (Figure 11) for MLD1‐CONST show the forcing for the cold‐hot‐warm, three‐band structure in the MLD1‐CONST experiment that was apparent in Figure 9, confirm that the cold SST anomalies over the driest tercile (TCWV percentile 0%–33%) is driven by enhanced OLR (Figure 11a), which is offset by warming due to increased clear‐sky SW radiation due to the reduced absorption by atmospheric water vapor (Figure 11c), SW cloud forcing (Figure 11d), and latent fluxes (Figure 10a). Meanwhile, the development of the band of warmest SSTs over the mid‐tercile of column water vapor values is due to enhanced downwelling SW in the absence of clouds in the clear moist columns (Figure 11d) and reduced outgoing LW flux. The cooling of SSTs in the moistest regions is dominated by the SW cloud forcing by the cirrus anvils and low cloud.

With the interactive lower boundary in MLD1‐CONST, the thermodynamic feedback (humidity and stability) on surface fluxes is considerably reduced in the vicinity of convection by around 4 W m^−2^, 20% of the original signal in MLD20‐CONST (Figure 6b). This is due to the tighter coupling of the SST and PBL properties with the thin mixed layer, and acts to oppose clustering by reducing the strength of the surface flux feedback near convection (Emanuel, 1987; Neelin et al., 1987). We note that the WISHE mechanism in hurricanes is explained in terms of convection‐induced velocity enhancements, whereas the sensitivity of the feedback to the ocean MLD is through the thermodynamic impact. While this is through stability in the pre‐onset, it is again the humidity differences between boundary and ocean that are important in the clustered phase (Figure S3b). This feedback would be much less sensitive in simpler bulk aerodynamic surface flux formulae that are still employed frequently in cloud resolving models and that neglect the impact of boundary layer stability on surface flux formulation.

**Figure 12:**
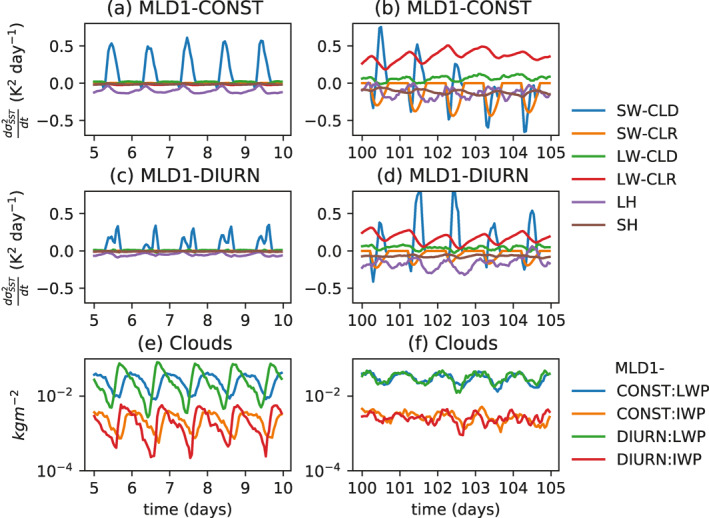
Contribution to SST variance from the LH, SH, SW, and LW cloud and clear‐sky surface components for experiments (a, b) MLD1‐CONST and (c, d) MLD1‐DIURN for days (a, c) day 5–10 and (b, d) day 100–105 of the simulations. It is recalled that the SST relaxation scheme only impacts the domain and thus does not affect SST variance. (e, f) The domain average total column liquid water path (LWP) and ice water path (IWP) in kg m^−2^ for the (e) pre‐onset day 5–10 and (f) clustered day 100–105 periods for both experiments.

While the hovmöller graphs show the general spatial forcing of SST anomalies, the drivers of SST variance for MLD1‐CONST are derived exactly in Figure 12 for a 5 day period, calculating the variance from the surface fluxes anomaly *F*′:

(4) $$\frac{\partial \sigma_{sst}^{2}}{\partial t}=\frac{2\bar{SST^{\prime }F^{\prime }}}{\rho_{l}C_{pl}h}.$$

In the clustered state (Figure 12b), the main driver of SST variance is confirmed to be the clear sky LW flux, with a weaker contribution from the LW cloud forcing. The SW cloud‐radiative forcing oscillates between forcing and damping of SST variance as convection migrates toward the warm SST zones, while the latent and sensible surface heat fluxes along with the SW clear sky fluxes are seen damp SST variability, confirming the interpretation of the hovmöller analysis (Figure 11).

**Figure 13:**
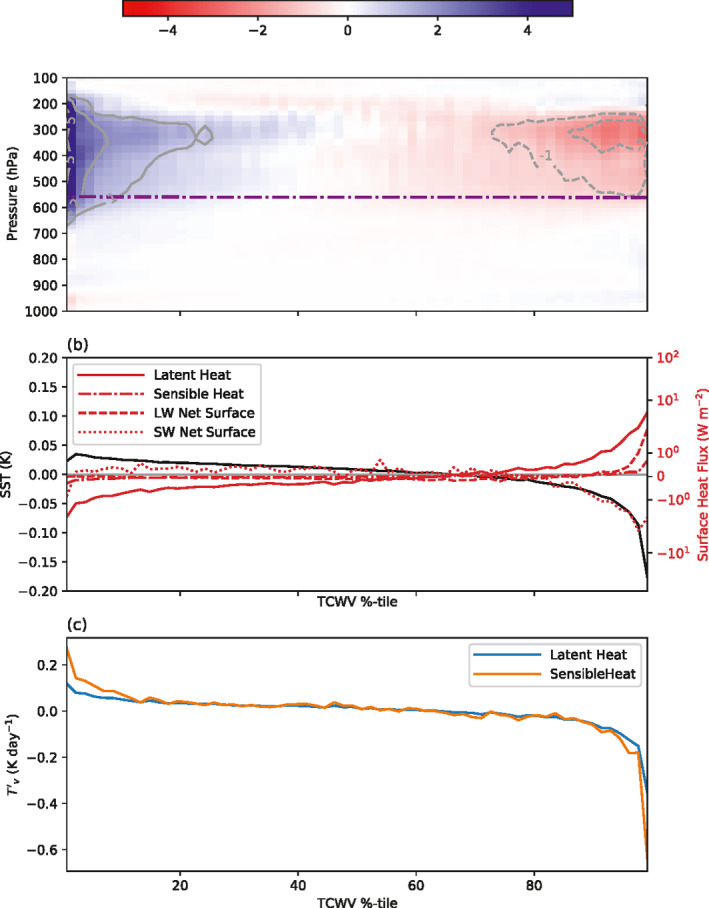
(a) Difference between Day 3–10 average of the MLD1‐CONST minus the MLD20‐CONST experiment member 0, normalized water vapor perturbation (color shading and gray contours). Purple dot‐dash line is the freezing level of MLD20‐CONST experiment. (b) Difference in SST (left) and surface fluxes (right axis, positive downwards) and (c) Difference in virtual temperature tendency in lowest model layer.

The SW cloud forcing of SST variance leads to the SST gradients in the early stages of the simulations that weakens the deep convective overturning circulation, resulting in a relative moist anomaly over the driest columns in MLD1‐CONST (Figures 13a and 13b). At the same time the gradient of latent and sensible heat fluxes contribution almost equally to the boundary layer buoyancy flux gradient (Figure 13c). As the thinner mixed layer can respond more rapidly to changing anomalies in SW forcing, the warmer SSTs under the driest columns in MLD1‐CONST enhance surface latent heat fluxes by increasing both the temperature and humidity differences between the ocean and atmosphere (Figures 6b and 13b), as well as the OLR emission. The gradient of the latent heat flux is on the order of 4 W m^−2^ and is a negative feedback that acts to slow clustering. Indeed, in the MLD1‐CONST experiment, the fast responding surface actually reverses the gradient of the latent heat flux such that it acts against clustering throughout the simulation, also in the pre‐onset phase (recall Figure 10a). This is also true for the buoyancy flux, where changes to both the sensible and latent heating act to increase the buoyancy flux in the driest region away from convection (Figure 13c), and was emphasized as important in the recent simulations of D. Yang (2018). The increased surface fluxes would also act to deepen the PBL in the dry regions, which leads to an OLR heating anomaly at the PBL top in the dry regions and a corresponding low level cooling in the moist regions. Converted into units of W m^−2^, this anomaly is similar in magnitude to the latent heat flux and would result in a low level circulation that would act to oppose clustering, relative to the 20 m mixed layer experiment. This mechanism involving SST gradients was previously suggested by Hohenegger and Stevens (2016). Thus to summarize, the enhanced downwelling shortwave flux in clear sky regions leads to a relative surface warming with thin mixed layers, while there is a relative surface cooling in cloudy regions (recall that the spatial mean is fixed). This results in a enhanced latent heat flux and reduced low cloud, both of which act to oppose clustering, delaying the onset, but once clustering gets underway the longwave emission to space dominates, leading to cold ocean temperatures in the dry regions.

**Figure 14:**
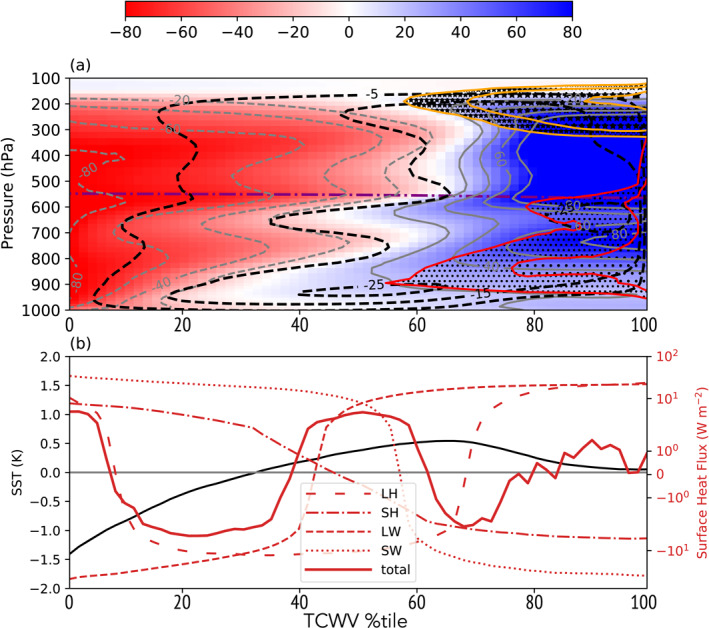
For experiment MLD1‐CONST Day 50–100 average of (a) normalized water vapor perturbation (color shading and gray contours), liquid water mixing ratio (dot shading and red contours marking 10^−5^, 3.10^−4^ and 10^−4^ kg kg^−1^) and ice water mixing ration (star shading and orange contours marking 10^−6^, 3.10^−5^ and 10^−5^ kg kg^−1^). The purple line marks the freezing level. (b) Surface fluxes (positive downwards) and SST anomalies.

The structure of the normalized water vapor perturbations and the mean cloud liquid and ice water in the clustered equilibrium state (day 50–100) of MLD1‐CONST is shown in Figure 14. The water vapor anomaly has a distinct mode‐2 structure (Mapes & Houze, 1995), with the strongest spatial anomalies occurring in the upper troposphere, between the mid‐level detrainment peak (Johnson et al., 1999) and the level of neutral buoyancy anvil top level (stars/orange contours). Comparing the water vapor and cloud structure (Figure 14a) to the SST and surface flux anomalies (Figure 14b), it is confirmed that the bands of warmest SSTs (that reside over the intermediate tercile of column water vapor) are associated with the areas of moist lower troposphere but dry upper troposphere, and are mostly clear sky or on the edge of the anvil outflow or shallow convection. The moist lower troposphere will prevent excessive loss of infrared radiation to space, while the clear sky and lack of cloud allow increased solar radiation to reach the surface and both the LW (red dashed) and SW (red dotted) flux anomalies contribute to the warm band. This evolution from the two zone to three zone cold‐hot‐warm SST structure was also documented in the simulations of Shamekh et al. (2020) using the SAM model.

The enhanced boundary layer enthalpy in the hot SST regions would be expected to lead to deep convection migrating constantly to this “border zone” between the moist and dry regions. The fact that the surface radiative and latent/sensible heat fluxes as a function of TCWV are locally non‐zero in the equilibrium states imply that the equilibrium must be a dynamic, non‐stationary state, in which convection migrates to hotter SST locations in which the SST then cools while the convection contemporaneously moistens the atmosphere in these locations. This action of convection migrating around inside a moist envelope is similar to the description of observations made by Blanco et al. (2019) and Mapes et al. (2018) and also the “cat and mouse” interpretation of convection and SST in simulations by Grabowski (2006).

At the same time, the large longwave atmospheric cooling over the moistest clear sky columns relative to the lower emissivity dry columns implies a similar dynamical state, whereby the driest columns in the domain, with lower emissivity and radiative cooling (as discussed at length in Emanuel et al., 2014) must moisten relative to the moist clear columns residing over the hottest SSTs. With the dry region developing and then moistening on a slow radiative overturning timescale of 20–30 days. We conjecture that the SST cold region “overshoot” and recovery noted in Figure 7 after clustering onset is the first wave of a 20–30 days oscillatory behavior in the clustered state driven by the water feedback. Computing resources limited these simulations to 100 days, only adequate to reveal the second oscillation toward the end of each experiment, but longer simulations are required to investigate this further.

**Figure 15:**
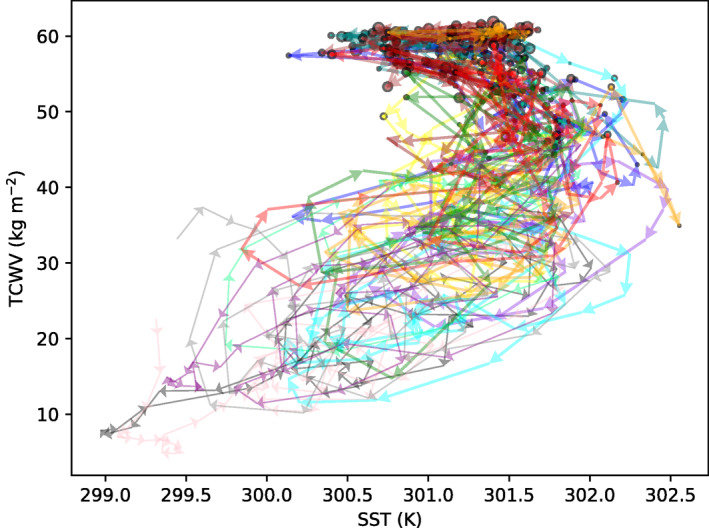
SST, TCWV, and cloud water paths are averaged over 16 × 16 km blocks and daily averaged from 00, 06, 12, and 18Z values, and 15 blocks are randomly chosen without replacement. The phase SST‐TCWV phase evolution of each block is illustrated with a different color for days 45–100 of the MLD1‐CONST experiment, member 0, with arrows indicating the direction of evolution. Color points are proportional to total column cloud water (ice + vapor), with a black point border indicating ice is present in the column. Thicker arrows indicate that TCWV exceeds 55 kg m^−2^ at some point in the 55 day period and thus the block is situated in a deep convective area at some point during the clustered state.

An attempt to illustrate the dynamical nature of the equilibrium state is illustrated in Figure 15, which divides up the domain into 16 × 16 km blocks and then plots the evolution of 15 randomly selected blocks in terms of the column humidity and the SST. The TCWV‐SST phase space shows a rotated V‐shape, with a negative correlation between SST and column humidity when TCWV exceeds approximately 50 kg m^−2^, in which all columns are cloudy. In this moist zone, the evolution of columns is dynamic, with SST evolution warming to as much as 302.5 K and then cooling to as little as 300 as convection occurs. In the lower “drier” branch of the phase diagram, while evolution is still chaotic, there is a general clockwise evolution of columns in the SST‐TCWV phase space, whereby columns with TCWV between 20 and 40 kg m^−2^ residing over the warmest SSTs undergo drying, which then leads to enhanced cooling by OLR emission, and a cooling of the surface temperatures. The driest columns instead have reduced subsidence rates and undergo moistening, with SST subsequently mostly rapidly warming once TCWV exceeds 35 kg m^−2^. Both the overturning exchange in the dry branch and the convective migration in the moist region would act to enhance water vapor bimodality and sharpen the gradient between the moist and dry regions (Zhang et al., 2003). This is clearly seen in Figure 14a (gray contours), where the water vapor gradients are very limited in the lower and upper TCWV terciles, and much stronger in the middle tercile.

## 5 Impact of Diurnal Cycle

In the previous section, we saw that the spatial variation of SST allowed by the implementation of a thin MLD weakened the diabatic forcing driving the tendency toward convective clustering, significantly delaying onset. Here, we now additionally allow the mean SST to respond to diurnal forcing and examine the impact on organizational onset using the OLR. We re‐emphasize that all experiments have a diurnal cycle in atmospheric radiative heating and the *spatial* forcing of the ocean. Thus the only difference in experiment MLD1‐DIURN is that the *domain mean* SST undergoes a diurnal cycle, while the *multi‐day, domain mean* SST is held exactly fixed in all experiments.

**Figure 16:**
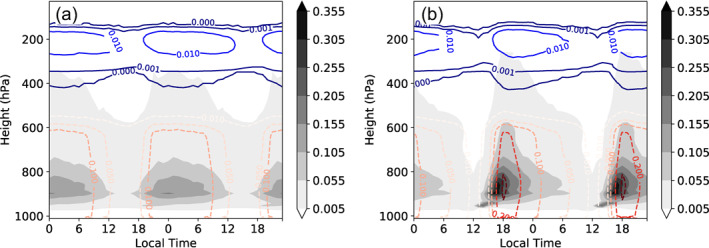
Diurnal cycle of domain means liquid cloud water (filled contours), ice water (solid contours), and total rain (dashed contours) for member 0 of the (a) MLD1‐CONST and (b) MLD1‐DIURN experiments. Units are kg kg^−1^ and zero represents local midnight. The mean daily cycle is repeated for clarity.

**Figure 17:**
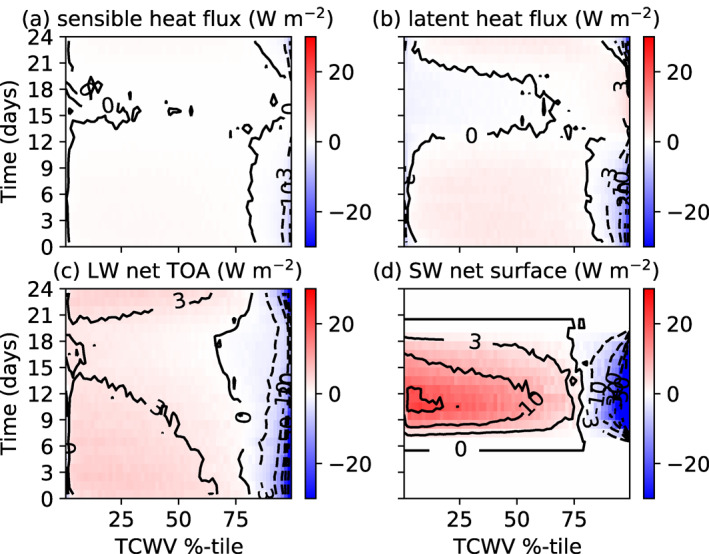
MLD1‐CONST day 3–10 mean diurnal cycle of flux perturbations ordered according to column water vapor for (a) surface sensible heat flux (positive downwards), (b) latent heat flux (positive downwards), (c) TOA LW flux (positive upwards), and (d) surface SW flux (positive downwards).

Observational studies have documented the diurnal cycle of convection in the tropics with generally a weak diurnal cycle with nocturnal to early morning peak over oceans (Albright et al., 1985; Bellenger et al., 2010; M. F. Cronin & McPhaden, 1999; Dai et al., 2007; Gray & Jacobson, 1977; Hall & Vonder Haar, 1999; Janowiak et al., 1994, 2005; R. J. Reed & Jaffe, 1981; Webster et al., 1996; G.‐Y. Yang & Slingo, 2001), but with convection over ocean shifting to a land‐like afternoon peak when calm, low wind conditions prevail which result in thin mixed ocean layers and significant diurnal surface heating (S. S. Chen & Houze, 1997). As the domain mean SST is constrained in MLD1‐CONST experiment, the cycle of convection is dominated by the atmospheric heating by shortwave absorption and resemble the maritime cycle over thick mixed layers. Convection occurs in a broad peak during night time through to the early morning hours when infrared cooling is not offset by SW absorption and instability is at a maximum (Figures 12e and 16a). The time‐mean spatial diurnal forcing of the ocean in the pre‐onset phase (day 3–10) in experiment MLD1‐CONST is clear in Figure 17d, with enhanced downwelling SW flux in the clear sky regions, and reduced in the cloudy moist columns. The net top‐of‐atmosphere LW flux instead follows the diurnal cycle of convection, with the maximum TOA cooling from clouds occurring in the early morning (Figure 17c). The surface sensible and latent heat fluxes also follow the convective diurnal cycle, with enhanced fluxes in the convective regions in the early morning, which were shown earlier to be the result of increased winds and reduced stability. The time series of spatial standard deviation of SST shows an interesting reversal in the spatial diurnal cycle in the pre and post onset phases (refer to Figure S5). In the pre‐onset, heterogeneity in solar fluxes acts to warm the SST in dry zones where the convection is suppressed, whereas after the onset of clustering and the formation of the very dry patch overlying colder SSTs due to excess OLR, the daytime heating anomaly in these regions acts to offset the OLR cooling and thus reduces spatial SST variance.

In the MLD1‐DIURN experiment, the domain means SST is unconstrained on daily timescales, and is subject to a cycle of 2.5 K in amplitude (see Figure S6), as expected for the one meter MLD. It exceeds slightly the observed cycle in low wind conditions in the West Pacific warm pool documented in Bernie et al. (2005) and Sui et al. (1997), but Bellenger et al. (2010) and Soloviev and Lukas (1997) showed that in calm conditions the diurnal warming of the upper 1 m of the ocean could reach 3 K. This diurnal forcing at the surface initially leads to a significant change in the characteristic of diurnal cloud variations, with the peak in rainfall shifting to the late afternoon at 4–6 p.m. local time (Figures 12e and 16b ). The rainfall peak is preceded by a deepening of the shallow cloud layer during the afternoon hours through to the congestus. The timing of this development appears to agree closely with the observations of convection in the Indian Ocean in weak wind conditions that lead to an ocean diurnal water layer. Bellenger et al. (2010) state that in these conditions a 3 p.m. peak in convection is associated with shallow systems, which then decrease while deep convection increases to peak in the late evening hours. The cycle is sharper, with greater maxima of precipitation and cloud liquid water and more exaggerated undulations in cold cloud top temperature relative to MLD1‐CONST. The afternoon convective peak has also been observed over the Pacific tropical convergence zone by Nitta and Sekine (1994), with S. S. Chen and Houze (1997) noting that the afternoon peak in convection was seen predominately in suppressed phases of the Madden‐Julian Oscillation, when winds are lighter and the diurnal SST variance is expected to be larger, similarly noted by Bellenger et al. (2010).

**Figure 18:**
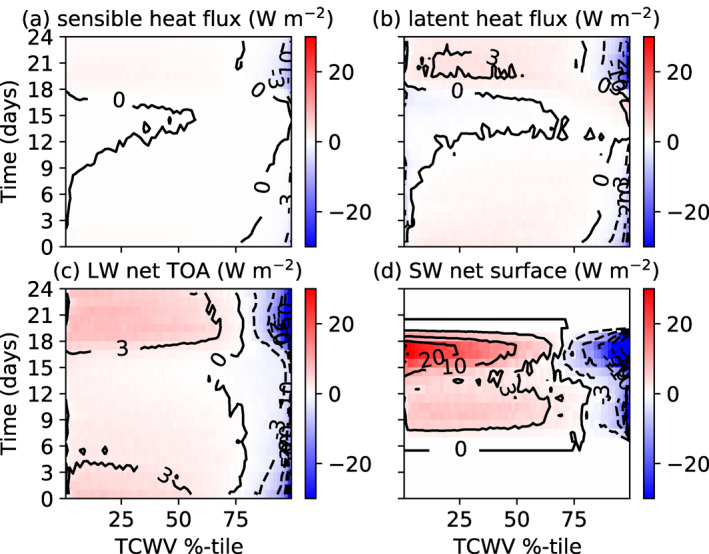
As Figure 17 for MLD1‐DIURN.

The shifted diurnal cycle of clouds impacts the diurnal cycle of the spatial gradients of fluxes (Figure 18). The surface latent and sensible heat fluxes spatial variations shift as expected to have the maximum differences between moist and dry regions at 6 p.m. local time, when deep convection peaks. This is also seen in the TOA LW fluxes subject to a slight lag associated with the convective development time, where the peak in cold cloud leads to a large reduction in net radiative flux over the moist columns at around 8–10 p.m. The SW surface flux shows a bi‐diurnal structure, with a maximum in the spatial flux anomalies during the local morning, which then reduce in tandem with cloud cover to reach a relative minimum at midday. The flux variance increases again during the afternoon with the growth of shallow convection, to reach a second maximum in the evening as deep convection initiates.

The source terms for SST variance due to each radiative flux component, divided into clear sky and cloud forcing, along with the surface latent and sensible heat fluxes are shown in Figure 12d. In the pre‐onset phase, the SW cloud forcing is seen to be much reduced in the MLD1‐DIURN experiment relative to MLD1‐CONST, due to the temporal shift in convective cloud activity. In MLD1‐DIURN cloud cover is at a relative minimum at mid‐day, reducing the forcing of SST variance. The resulting reduction in SST variance would weaken the feedback of SST variability. The reduction in the spatial SST gradients would be expected to result in clustering occurring earlier in the MLD1‐DIURN experiment. In Table 2, it is seen that this is the case for the two OLR‐based TOC metrics, with organization occurring about 1.5 days earlier in MLD1‐DIURN, but with onset actually about 3 days later using *I*_*org*_. However, these differences are small when compared to the standard deviation in onset within the six member ensemble, with an ensemble spread of approximately 5–6 days for MLD1‐CONST and 9–10 days for MLD1‐DIURN, according to the TOC metric used. Using a two sample Student's *T* test, this difference in onset time between the two experiments is not statistically significant, even at the 80% level. The OLR timeseries for each of the six ensemble members (Figure 8) reiterates the strong variability between the experiments, and lack of a significant shift in onset time. This result highlights the importance of the carefully designed experiment that ensures domain mean SSTs are exactly controlled, to ensure that the impact of drifting mean SSTs is not confounded with their diurnal variations. It also emphasizes the importance of conducting ensembles of simulations to ensure impacts on clustering onset time can be isolated from stochastic effects.

Why is the reduction in the spatial SST gradients not having a greater impact on the clustering onset time? One reason could be related to cold pools and the sharpness of the diurnal cycle. In simulations, deep convection is mutually exclusive and regularly spaced (that is, clouds are further apart on average than would be expected by a random process) at scales below around 15–20 km, attributed to the role of cold pools (Tompkins & Semie, 2017). Modeling studies have also demonstrated how the suppression of cold pool activity leads to rapid aggregation onset (Jeevanjee & Romps, 2013; Muller & Bony, 2015).

**Figure 19:**
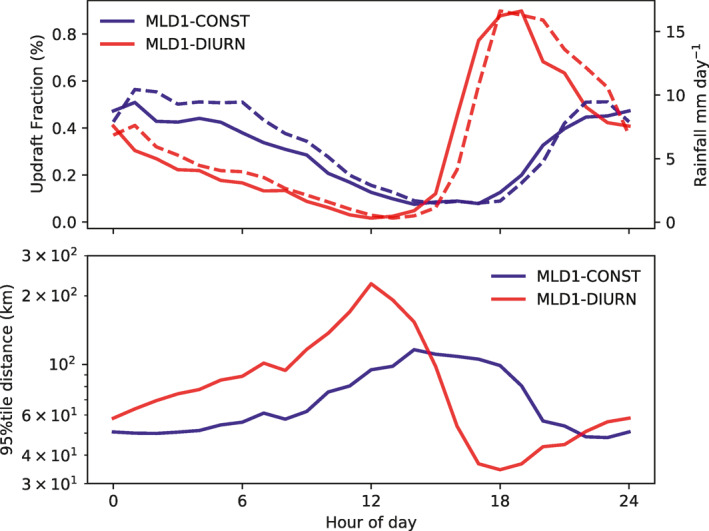
Timeseries of the mean diurnal cycle of (a) Convective updraft fraction (cells with *w* > 1 m s^−1^ at 700 hPa, solid lines, left axis), and precipitation (dashed lines, right axis) and (b) the 95% tile of the distance to the nearest convective point (km), *d*_95_ for the MLD1‐CONST and MLD1‐DIURN, experiments.

Figure 19 compares the mean diurnal cycle in the pre‐onset phase (day 5–10) in MLD1‐DIURN and MLD1‐CONST of the convective updraft fraction (number of cells with *w* > 1 m s^−1^ at *p* ≈ 700 hPa), surface rainfall (Fig. 19a), and *d*_95_, the 95th percentile of the distribution of distances to the nearest convective updraft in km. Rainfall closely tracks the updraft fraction with a small lag of one hour (the temporal resolution of the analysis) as expected (Figure 19a). Indeed, the profiles scale linearly, which indicates that the increases in rainfall occur almost entirely from increases in the number of separate convective events (updraft core diameter is approximately invariant) and not through the intensity of rainfall per convective core. The strong diurnal cycle in domain‐mean rain rate thus implies that during the afternoon peak there are more cells present, and a consequence of this is that the afternoon minimum between hour 15 and 18 in *d*_95_ is lower in the stronger diurnal case. Thus, as the convective diurnal cycle is sharper in the pre‐clustered phase, convection covers a larger portion of the domain due to the mutual exclusivity caused by cold pools, distributing the moisture sources more widely over the domain. It is the distribution of moisture sources of convection that is key during the time of the day when convection is most active and *d*_95_ is at a minimum, as it is at this time that the major input of moisture to the free troposphere occurs in the form of convective detrainment.

One unexpected finding of the MLD1‐DIURN simulations is that the late afternoon peak in the convective diurnal cycle is only sustained in the pre‐clustered, random convection stage. Once the clustering onset starts, the diurnal cycle weakens and shifts back to an early morning maximum, identical to the MLD1‐CONST experiment (Figure 12 panel f). This reversion of the diurnal cycle could also contribute to the organization onset times being similar.

The SST and boundary layer moist static energy still maintain a diurnal cycle in the organized state with a similar magnitude and phase, and thus the lapse rate forcing of the diurnal cycle is also unchanged. We note that the lapse rate forcing in Ruppert and Hohenegger (2018) only referred to the free troposphere cooling, as SST was kept fixed in their simulations, and would contribute to an early morning maximum. Here the lapse rate impact is dominated by the PBL moist static energy which would indicate a late‐afternoon precipitation maximum.

**Figure 20:**
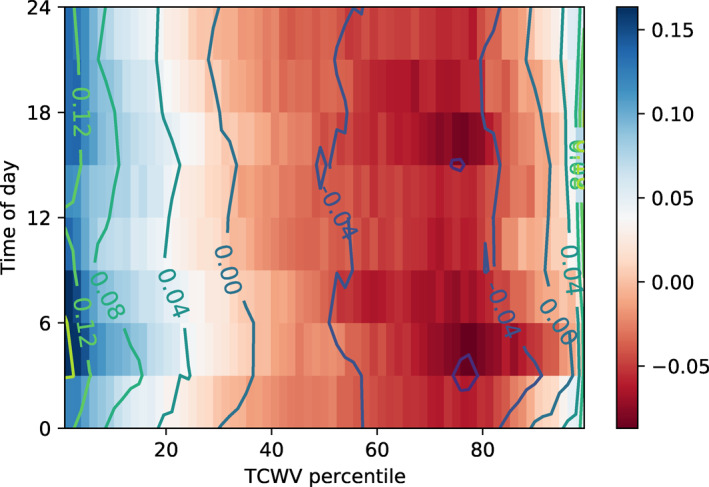
Diurnal cycle of pressure perturbation in the lowest model level sorted according to the TCWV percentile in experiment MLD1‐DIURN. Similar perturbations are seen throughout the PBL, while in the free troposphere the diurnal variations are minimal.

**Figure 21:**
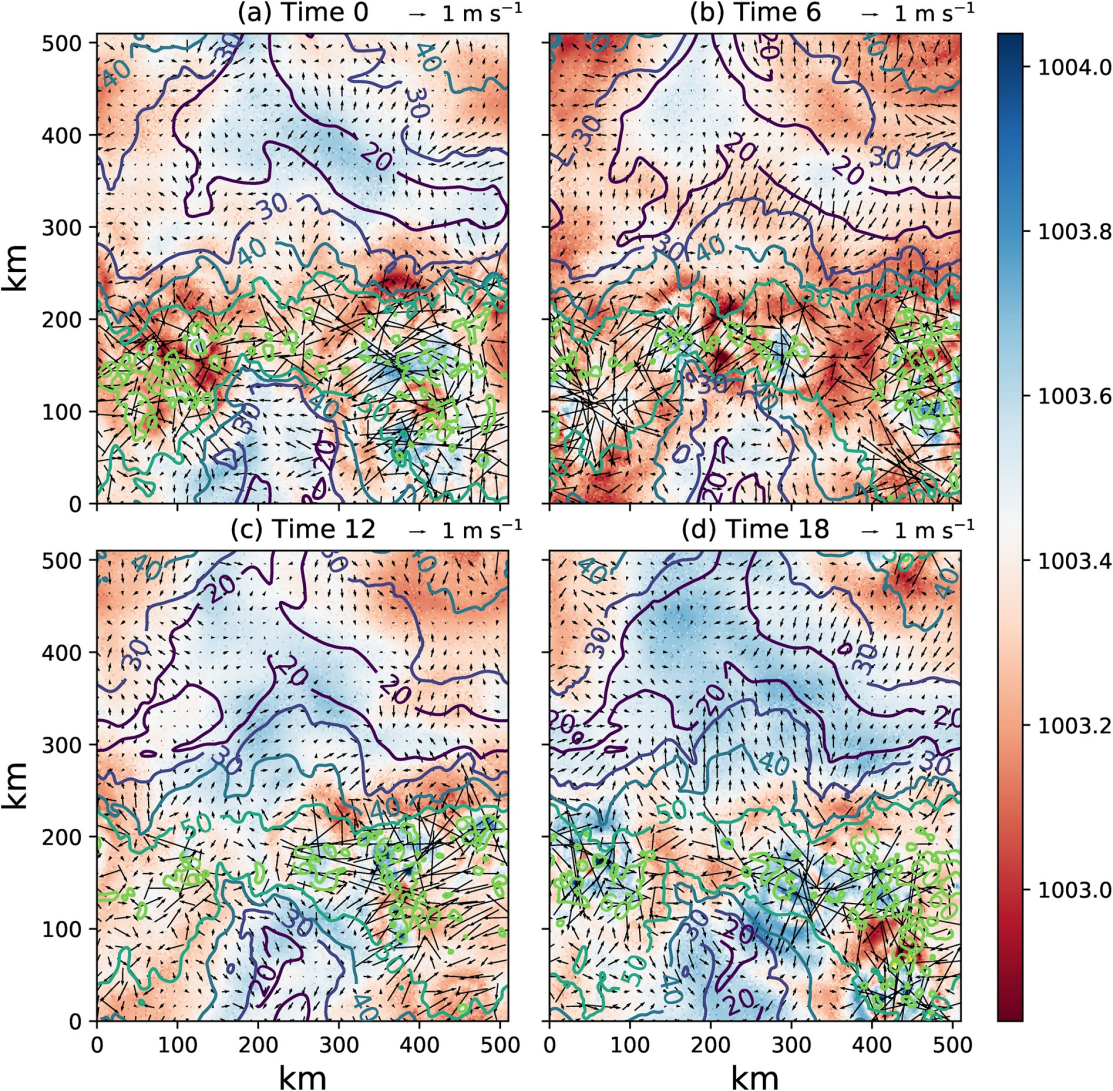
Maps of surface pressure (hPa) in experiment MLD1‐DIURN, with contours showing the TCWV (kg m^−2^) and wind vectors showing the *anomalous* wind around the day mean (see vector key for magnitude). Figure panels are for day 100 (a) hour 0, (b) hour 6, (c) hour 12, and (d) hour 18.

Instead, the shift in the diurnal cycle is due to radiative impact of the dry columns and the heterogeneity in the heating rates, a mechanism related to that described in Gray and Jacobson (1977). At night, there is increased net radiative cooling in the boundary layer in these regions due to the presence of the very dry mid to upper troposphere. This leads to an enhanced pressure gradient in the boundary layer in the early hours of the morning (Figure 20). The pressure gradient drives an anomalous low level circulation with anomalous convergence into moist regions at 6 a.m. local time, while at 6 p.m. there is anomalous divergence from the moist to the dry regions (Figure 21). This feature of the enhanced cooling in dry regions was termed radiatively driven mesoscale coldpools by Coppin and Bony (2015), and the boundary layer pressure gradients and anomalous flow is similar to that documented in Naumann et al. (2017). Thus, even though the moist static energy and CAPE still peak in the late afternoon, the spatial heterogeneities in the net heating rates due to water vapor (since cloud radiative forcing is just as strong in the pre‐onset phase) dominate the diurnal cycle.

If these model results for this idealized experimental framework are indicated of tropical convection, it would indicate that low wind, suppressed conditions, are a necessary but non‐sufficient condition for the diurnal cycle to exhibit a late afternoon maximum. In addition, spatial heterogeneity in water vapor would need to be limited. In the model's clustered state, spatial water vapor heterogeneity is such that a water vapor range of 20–70 kg m^−2^, values that are often observed in the western Pacific warm pool region, was sufficient to shift to a weak morning diurnal peak.

## 6 Conclusions

As the degree of organization or “clustering” of convection can have a significant impact on the tropical radiative budget, it is important to understand its origin. In the past, much has been gained from conducting idealized experiments in homogeneous environments with convection permitting models, which have shown how convection‐cloud‐vapor‐radiation interactions can lead to strong clustering of convection in these highly idealized frameworks. Here, in the spirit of systematically adding to model complexity one process at a time, we have implemented a slab ocean model to allow the surface to interact with the overlying atmosphere, first allowing it to only respond to fluxes spatially, and then also in the domain‐mean in response to solar diurnal forcing. Since previous work has demonstrated potentially strong sensitivities of clustering to mean SST, the framework for the experiment was carefully designed to divide the slab ocean response between spatial feedbacks and feedbacks involving the mean diurnal cycle in SST, while ensuring that any drift in the long term mean SST was completely absent from all experiments.

In the first experiment, where the domain‐mean SST is constant and only spatial feedbacks are permitted, the diurnal cycle in atmospheric absorption of solar radiation results in a weak maritime‐like diurnal variation in convective activity, with the maximum occurring during the night and early morning hours. In this framework, the interactive ocean delayed the onset of convective clustering, with the delay increasing as the MLD of the ocean decreases. With a thin, one meter mixed layer, the onset time was almost doubled from 21.7–27.5 to 36.9–45.7 days (with the range due to different metrics of onset). The contrast in surface solar radiation between cloudy and clear sky regions results in warmer temperatures (with differences of up 0.35 K). This leads to enhanced surface latent and sensible heat fluxes in the clear sky regions away from convection, impacting the boundary layer moist static energy and buoyancy gradients, which would reduce the low level pressure gradient and convergence into the convective regions, as also documented by Shamekh et al. (2020).

Once clustering occurs, the strong column drying leads to excessive loss of OLR and the formation of a cold ocean patch, over which convection and cloud are absent. The domain then clearly divides into three distinct zones, this cold‐dry zone, an intermediate boundary zone of moist clear sky air, and then the convective cloudy zone. The absence of anvil cloud in the intermediate moist‐clear region, and the high humidity, leads to the domination of the SW anomaly over the longwave, and the high SSTs occur in this region. Thus convection constantly migrates into this changing boundary zone over the warmest SST, but can not enter the neighboring dry zone. It was suggested that this is reminiscent of convection moisture structures in the tropics, and this mechanism could be part of the origin of upper tropospheric moisture bimodality which will be investigated further.

In the second experiment, the mean SST is unconstrained on diurnal time scales, but all drift in the long‐term SST is absent using a new slaved target SST method. Over thin 1 m ocean mixed layers, the strong diurnal cycle in domain‐mean SST lead to the convection peak shifting to early evening in the early stages of the simulations when convection is still random; agreeing with observations over tropical oceans that occurs in weak wind, “suppressed” conditions. The resulting greater day‐time anvil cloud coverage reduces the solar‐driven heterogeneity in spatial SST variance. This would in theory act to encourage earlier onset of clustering, but is offset by the tighter diurnal cycle resulting in more evenly spaced moisture sources due to the inhibiting role of convective cold pools. Both these effects reduce in magnitude as clustering starts and convection reverts to a weak early morning peak despite the persisting diurnal surface forcing. This shift is the result of the differential radiative heating contrasts between moist and dry areas, which drives anomalous convergence into the moist regions in the night hours. Thus it appears that if spatial gradients of humidity are sufficient, radiative heterogeneity can dominate the thermodynamic forcing of the diurnal cycle through the local lapse rate. This implies that thin mixed ocean layers present in suppressed conditions are a necessary but non‐sufficient condition for an afternoon maximum of convection in maritime regions. In addition, spatial heterogeneity of water vapor would need to be limited to prevent spatial contrasts in radiative heating driving an early morning maximum. The result is that the mean onset time of clustering, while slightly earlier in the diurnal experiment was not statistically different from the experiment with no diurnal cycle in domain temperatures, highlighting the importance of conducting ensembles of simulations to gauge stochastic variability.

## Supporting information

Supporting Information S1

Figure S1

Figure S2

Figure S3

Figure S4

Figure S5

Figure S6

## Data Availability

Key model output from the six member ensemble experiments are freely available in netcdf format at http://clima-dods.ictp.it/Users/tompkins/data/wrf/ and will be maintained for a minimum period of 5 yr from the date of publication, along with the neural network model used for surface fluxes.
