# Supplementary material for: Impact of a Mixed Ocean Layer and the Diurnal Cycle on Convective Aggregation
Source: J Adv Model Earth Syst. 2021 Nov 25;13(12):e2020MS002186. doi: 10.1029/2020MS002186 (PMC9285392; doi:10.1029/2020MS002186)
Supplement: Supplementary file 1 — Supporting Information S1 [file JAME-13-0-s003.pdf]

Supporting Information for "Organization of convection in low vertical wind shears: impact of interactive ocean and diurnal cycle"

Adrian M. Tompkins [1],Addisu G. Semie[2,3]

[1]Earth System Physics, Abdus Salam International Centre for Theoretical Physics (ICTP), Trieste, Italy

[2]Laboratoire de Meteorologie Dynamique (LMD/IPSL), Sorbonne University, CNRS, Paris, France

[3]Computational Data Science Program, Addis Ababa University, Addis Ababa, Ethiopia

Contents of this file

1. Figures S1 to S6

Introduction

This file contains a text overview of the experimental set up and parameterization choices, a summary figure for the performance of the neural network flux proxy model, and a figure illustrating how the adaptive Q-flux boundary condition allows a full diurnal cycle to be represented while removing any mean drift. Additional figures provide more supplementary information on the diurnal cycle and long-term evolution of the spatial mean and variance of the SST and well as the long term evolution of its variance.

Figure S1. Offline integration of a toy slab ocean model with a mixing layer depth h=1 meter, and the seawater heat capacity of c_{pl}=3850 J kg^{-1} K^{-1} and density ρ_l=1020 kg m^{-3}. Three comparison methods are shown to control the SST, to illustrate how the new

adaptive SST control mechanism permits a full diurnal cycle while avoiding SST drift from the desired boundary condition, set to $SST_b = 28^o$C as in the CRM simulations conducted in this paper. In panel a, an artificial surface net energy flux is constructed according to $F = Acos((2t+1)\pi) + I$, where A is the flux amplitude set to 300 W m^{-2} here, t is the time in days and I is the flux imbalance, which is set to an illustrative value of 120 W m^{-2}, marked by the dashed line. In panel b, three simulations of the SST are made, all using a relaxation timescale τ of 24 hours to allow a diurnal cycle. SST-relax shows the resulting SST using a classic Newtonian relaxation where $dSST/dt = (SST_t - SST)/\tau$, and SST_t is simply set to the target boundary temperature of SST_b. In this case, the SST follows a diurnal cycle, but the SST drifts by $\frac{\tau I}{C_{pl}\rho_l h}$, more than 2K. In SST-offset, a modified value $SST_t = SST_b - \frac{\tau \tilde{I}}{C_{pl}\rho_l h}$ is used, using an (incorrect) estimate of the imbalance $\tilde{I}$, which we set to 70 W m^{-2} for illustrative purposes. This reduces the drift but does not eliminate it due to the error in $\tilde{I}$ (note that I would be constantly evolving with the model mean state). In SST-adaptive, a second prognostic equation is introduced $dSST_t/dt = (SST_b - \overline{\overline{SST}})/\tau_{adj}$ (see paper for more details), with $\overline{\overline{SST}}$ the running mean of SST calculated using Welford's algorithm with a window of one day and τ_{adj}=2 days. Here the SST_t, shown by the red line, undergoes a slightly under-damped second order response and brings the SST to equilibrium by day 10 with an error<0.01 K in the mean temperature with respect to SST_b shown with the dashed line. Panel (c) is a repeat of panel (b) but with τ=12 hours, as used in this paper. This has the advantage of resulting in a faster adjustment to equilibrium and smaller drift in the first 3 days, but at the cost of a small damping of the diurnal cycle amplitude by about 4%.

Figure S2. Scatter plot of predicted surface latent heat flux from the Monin-Obukhov (M-O) scheme used in WRF and the neural network fit for a subset of 2000 randomly sub-sampled points from the validation dataset of $0.2*2^{18}$ data points, with flux plotted as a function of (top-left) Surface velocity, (top-right) Δq (mixing ratio difference between surface and lowest model level) (lower left) ΔT (temperature difference between surface and two meter level. The lower right panel plots the WRF fluxes directly against the neural network model.

Figure S3. Contributions to the latent heat surface flux perturbations during day 100-105 in the pre-onset phase of (a) MLD20-CONST and (b) MLD1-CONST minus MLD20-CONST differences, from wind perturbations, humidity (Δq) and stability (ΔT) differences, where Δq is the difference between the saturated mixing ratio at the sea surface and the mixing ratio of the lowest model level, and ΔT is calculated as the difference between the SST and the two meter temperature. Thermodynamic perturbations refers to the contribution from ΔT, Δq and the $\Delta T \Delta q$ terms. Calculations are made from a neural network fitted to the WRF full flux calculations (see methods for more details).

Figure S4. Daily mean spatial anomaly of SST arranged as a function of TCWV percentile for member 0 of the MLD1-CONST experiments. Columns are arranged in order and then an average of 1024 columns is applied (no block averaging). The two level stippled (hashed) contours show areas where the column mean liquid (ice) cloud water exceeds the thresholds of 0.01 (0.003) and 0.03 (0.01) kg m^{-2}, respectively with black (blue) colors. Red contours indicate rain rates of 10 and 25 mm day^{-1}. Note that instantaneous fields are sampled 4 times a day.

Figure S5. Timeseries of the spatial standard deviation of the SST for member 3 and 4 of the (a) MLD-CONST, (b) MLD1-DIURN simulations and (c) the mean of the two ensemble members for each experiment. In the pre-onset, the MLD1-DIURN has a reduced diurnal cycle due to the fact that the convection occurs predominately during the afternoon, thus greater anvil shielding reduces the spatial heterogeneity of the SW surface flux relative to MLD-CONST, where convection occurs around the clock with a weaker nocturnal diurnal maximum. Only two members are shown to illustrate the cancellation of the diurnal cycle of variance that occurs during the clustering onset when the mean of the two is calculated (deep blue lines), which is due to the cycle reversing. In the pre-onset, solar heating causes the surface to be warmest in cloud free areas, leading to a variance maximum during the day. After clustering onset, the SST cools substantially under the dry patch and in this phase the day time heating offsets this and leads to a variance minimum during the day. The graph also reveals interesting long term oscillations in the SST variance and a reduction in the diurnal cycle.

Figure S6. Timeseries of the diurnal variation of the (a) the spatial mean of SST, $\overline{SST}$ and (b) spatial standard deviation of SST, $\sigma(SST)$ averaged over day 5 to day 10 of the MLD1-CONST and MLD1-DIURN experiments.

Text S1: Experimental set up

Regarding the experiment set up, the domain size is 512 by 512 km, similar to Bretherton, Blossey, and Khairoutdinov (2005) with periodic lateral boundary conditions. Muller and Held (2012) found self-aggregation required domain sizes larger than 200 km although Tompkins and Craig (1998) documented strong aggregation even with 100 km length 3D domains, indicating a strong dependence of this threshold on model configu-

ration. Wing and Cronin (2016) introduced a theory for the spacing between convective clustered in organized states based on the boundary layer recovery that would suggest a single convective cluster is expected if convection organizes in a domain of this size. All model simulations use a 2 km horizontal spacing (as used by Tompkins & Craig, 1998; Bretherton et al., 2005; Muller & Held, 2012) and no convective parameterization scheme is used. A stretched vertical grid divides the atmosphere up into 62 layers. Domain-mean horizontal winds are constrained to be close to zero using a horizontally-homogeneous Newtonian relaxation term. This prevents the development of wind-shear during the simulation, as observed in previous simulations (Held et al., 1993; Tompkins & Craig, 1998; Tompkins, 2000), but does not impact the nature of convection or sub-domain circulations. In order to initiate convection in these simulations, a random perturbation is applied to the potential temperature field with amplitude of 0.1 K in the lowest level and linearly decreasing to 0.02 K in the fifth level.

Concerning the physical parameterizations used, the 5th order advection scheme is employed for both momentum and scalar horizontal fields (Skamarock et al., 2008) and the microphysics is parameterized using the Purdue Lin scheme that includes six classes of hydrometeers (water vapor, cloud water, rain, cloud ice, snow and graupel) (Lin et al., 1983). Gravity waves are damped at the domain top using Rayleigh damping to prevent unphysical wave reflection off the domain upper boundary (Skamarock et al., 2008), and rotation is absent. The radiative transfer scheme RRTMG calculates short wave ($RRTMG\_SW$) (Mlawer et al., 1997) and long wave ($RRTMG\_LW$) (Mlawer & Clough, 1997; Mlawer et al., 1997) fluxes and heating/cooling rates using the correlated k approach. The diurnal

cycle is included, with the solar declination calculated assuming a nominal location of 0° longitude and 10° latitude.

All simulations use the 3D Smagorinsky mixing for horizontal transport, while the YSU PBL scheme handles all subgrid-scale vertical transport. These simulations have the 6th order noise filter without enforcing monotonicity. It was noted in Tompkins and Semie (2017) that the various commonly employed turbulence schemes vary hugely in the degree of mixing they diagnose, which in turn had a critical impact on the occurrence of convective clustering. We have employed a configuration similar to those experiments in Tompkins and Semie (2017) that showed a strong propensity to undergo clustering.

References

Bretherton, C. S., Blossey, P. N., & Khairoutdinov, M. (2005). An energy-balance analysis of deep convective self-aggregation above uniform SST. *J. Atmos. Sci.*, *62*, 4273–4292.

Held, I. M., Hemler, R. S., & Ramaswamy, V. (1993, November). Radiative-Convective Equilibrium with Explicit Two-Dimensional Moist Convection. *Journal of the Atmospheric Sciences*, *50*(23), 3909–3927. doi: 10.1175/1520-0469(1993)050⟨3909:RCEWET⟩2.0.CO;2

Lin, Y.-L., Farley, R. D., & Orville, H. D. (1983, June). Bulk Parameterization of the Snow Field in a Cloud Model. *Journal of Climate and Applied Meteorology*, *22*(6), 1065–1092. doi: 10.1175/1520-0450(1983)022⟨1065:BPOTSF⟩2.0.CO;2

Mlawer, E. J., & Clough, S. A. (1997). Shortwave and longwave enhancements in the Rapid Radiative Transfer Model. In *Proc. of the 7th Atmospheric Radiation Measurement (ARM) Science Team Meeting* (p. CONF-9603149). U.S. Department

of Energy, available at http://www.arm.gov/publications: ARM.

Mlawer, E. J., Taubman, S. J., Brown, P. D., Iacono, M. J., & Clough, S. A. (1997). Radiative transfer for inhomogeneous atmospheres: RRTM, a validated correlated-k model for the longwave. *J. Geophys. Res.*, *102*, 16663–16682.

Muller, C. J., & Held, I. M. (2012, April). Detailed Investigation of the Self-Aggregation of Convection in Cloud-Resolving Simulations. *Journal of the Atmospheric Sciences*, *69*(8), 2551–2565. doi: 10.1175/JAS-D-11-0257.1

Skamarock, W. C., Klemp, J. B., Dudhia, J., Gill, D. O., Barker, D. M., Duda, M. G., ... Powers, J. G. (2008). *A Description of the Advanced Research WRF Version 3* (Vol. x; Tech. Rep.). NCAR.

Tompkins, A. M. (2000). The impact of dimensionality on long-term cloud resolving model simulations. *Mon. Wea. Rev.*, *128*, 1521–1535.

Tompkins, A. M., & Craig, G. C. (1998). Radiative-convective equilibrium in a three-dimensional cloud ensemble model. *Q. J. R. Meteorol. Soc.*, *124*, 2073–2097.

Tompkins, A. M., & Semie, A. G. (2017). Organization of tropical convection in low vertical wind shears: Role of updraft entrainment. *J. Adv. Mod. Earth Sys.*, http://dx.doi.org/10.1002/2016MS000802. doi: 10.1002/2016MS000802

Wing, A. A., & Cronin, T. W. (2016). Self-aggregation of convection in long channel geometry. *Q. J. R. Meteorol. Soc.*, *142*(694), 1–15, doi 10.1002/qj.2628.

Figure S1. Offline integration of a toy slab ocean model with a mixing layer depth h=1 meter, and the seawater heat capacity of c_{pl}=3850 J kg^{-1} K^{-1} and density ρ_l=1020 kg m^{-3}. Three comparison methods are shown to control the SST, to illustrate how the new adaptive SST control mechanism permits a full diurnal cycle while avoiding SST drift from the desired boundary condition, set to $SST_b = 28^o$C as in the CRM simulations conducted in this paper. In panel a, an artificial surface net energy flux is constructed according to $F = Acos((2t+1)\pi) + I$, where A is the flux amplitude set to 300 W m^{-2} here, t is the time in days and I is the flux imbalance, which is set to an illustrative value of 120 W m^{-2}, marked by the dashed line. In panel b, three simulations of the SST are made, all using a relaxation timescale τ of 24 hours to allow a diurnal cycle. SST-relax shows the resulting SST using a classic Newtonian relaxation where $dSST/dt = (SST_t - SST)/\tau$, and SST_t is simply set to the target boundary temperature of SST_b. In this case, the SST follows a diurnal cycle, but the SST drifts by $\frac{\tau I}{C_{pl}\rho_l h}$, more than 2K. In SST-offset, a modified value $SST_t = SST_b - \frac{\tau \tilde{I}}{C_{pl}\rho_l h}$ is used, using an (incorrect) estimate of the imbalance $\tilde{I}$, which we set to 70 W m^{-2} for illustrative purposes. This reduces the drift but does not eliminate it due to the error in $\tilde{I}$ (note that I would be constantly evolving with the model mean state). In SST-adaptive, a second prognostic equation is introduced $dSST_t/dt = (SST_b - \overline{\overline{SST}})/\tau_{adj}$ (see paper for more details), with $\overline{\overline{SST}}$ the running mean of SST calculated using Welford's algorithm with a window of one day and τ_{adj}=2 days. Here the SST_t, shown by the red line, undergoes a slightly under-damped second order response and brings the SST to equilibrium by day 10 with an error<0.01 K in the mean temperature with respect to SST_b shown with the dashed line. Panel (c) is a repeat of panel (b) but with τ=12 hours, as used in this paper. This has the advantage of resulting in a faster adjustment to equilibrium and smaller drift in the first 3 days, but at the cost of a small damping of the diurnal cycle amplitude by about 4%.

Figure S2. Scatter plot of predicted surface latent heat flux from the Monin-Obukhov (M-O) scheme used in WRF and the neural network fit for a subset of 2000 randomly sub-sampled points from the validation dataset of $0.2*2^{18}$ data points, with flux plotted as a function of (top-left) Surface velocity, (top-right) Δq (mixing ratio difference between surface and lowest model level) (lower left) ΔT (temperature difference between surface and two meter level. The lower right panel plots the WRF fluxes directly against the neural network model.

Figure S3. Contributions to the latent heat surface flux perturbations during day 100-105 in the pre-onset phase of (a) MLD20-CONST and (b) MLD1-CONST minus MLD20-CONST differences, from wind perturbations, humidity (Δq) and stability (ΔT) differences, where Δq is the difference between the saturated mixing ratio at the sea surface and the mixing ratio of the lowest model level, and ΔT is calculated as the difference between the SST and the two meter temperature. Thermodynamic perturbations refers to the contribution from ΔT, Δq and the $\Delta T \Delta q$ terms. Calculations are made from a neural network fitted to the WRF full flux calculations (see methods for more details).

Figure S4. Daily mean spatial anomaly of SST arranged as a function of TCWV percentile for member 0 of the MLD1-CONST experiments. Columns are arranged in order and then an average of 1024 columns is applied (no block averaging). The two level stippled (hashed) contours show areas where the column mean liquid (ice) cloud water exceeds the thresholds of 0.01 (0.003) and 0.03 (0.01) kg m^{-2}, respectively with black (blue) colors. Red contours indicate rain rates of 10 and 25 mm day^{-1}. Note that instantaneous fields are sampled 4 times a day.

Figure S5. Timeseries of the spatial standard deviation of the SST for member 3 and 4 of the (a) MLD-CONST, (b) MLD1-DIURN simulations and (c) the mean of the two ensemble members for each experiment. In the pre-onset, the MLD1-DIURN has a reduced diurnal cycle due to the fact that the convection occurs predominately during the afternoon, thus greater anvil shielding reduces the spatial heterogeneity of the SW surface flux relative to MLD-CONST, where convection occurs around the clock with a weaker nocturnal diurnal maximum. Only two members are shown to illustrate the cancellation of the diurnal cycle of variance that occurs during the clustering onset when the mean of the two is calculated (deep blue lines), which is due to the cycle reversing. In the pre-onset, solar heating causes the surface to be warmest in cloud free areas, leading to a variance maximum during the day. After clustering onset, the SST cools substantially under the dry patch and in this phase the day time heating offsets this and leads to a variance minimum during the day. The graph also reveals interesting long term oscillations in the SST variance and a reduction in the diurnal cycle.

Figure S6. Timeseries of the diurnal variation of the (a) the spatial mean of SST, $\overline{SST}$ and (b) spatial standard deviation of SST, $\sigma(SST)$ averaged over day 5 to day 10 of the MLD1-CONST and MLD1-DIURN experiments.
