# Supplementary figures and images for: Impact of a Mixed Ocean Layer and the Diurnal Cycle on Convective Aggregation
Source: J Adv Model Earth Syst. 2021 Nov 25;13(12):e2020MS002186. doi: 10.1029/2020MS002186 (PMC9285392; doi:10.1029/2020MS002186)

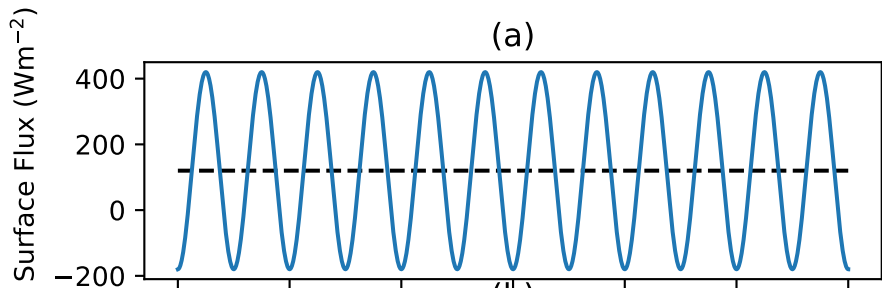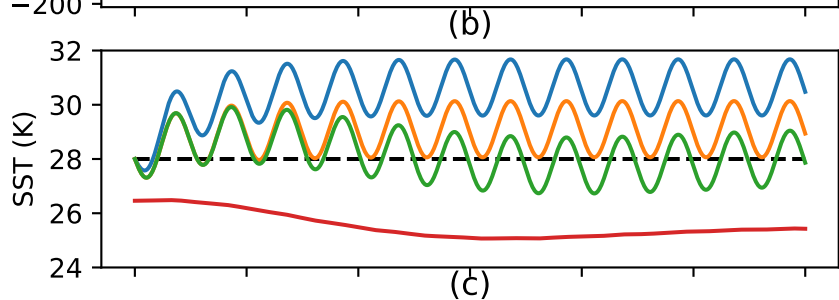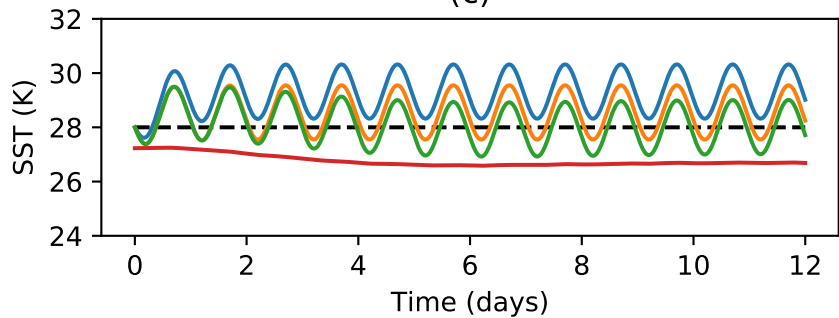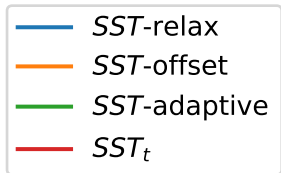

Supplement: Supplementary file 2 — Figure S1 [file JAME-13-0-s006.pdf]

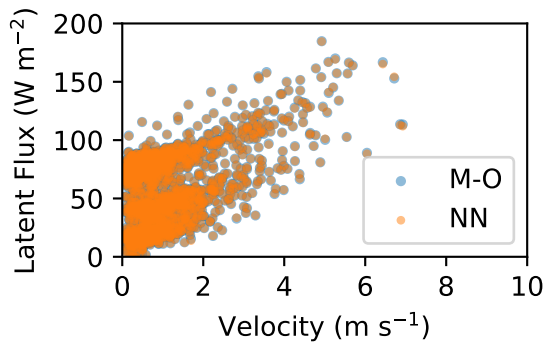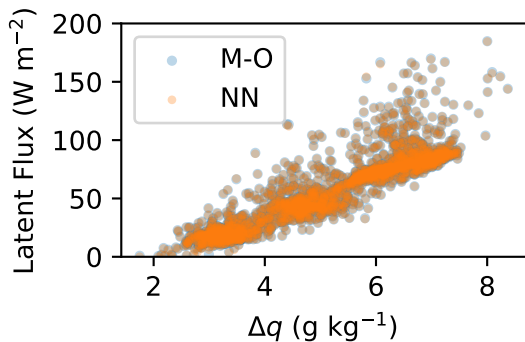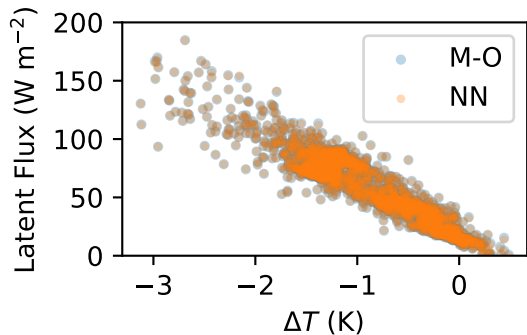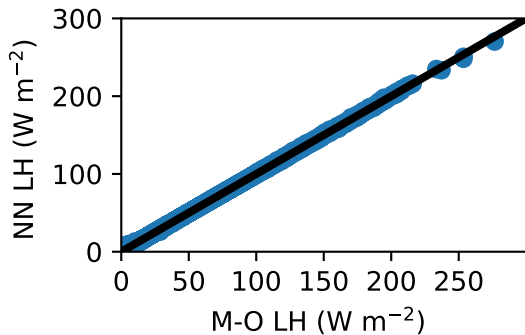

Supplement: Supplementary file 3 — Figure S2 [file JAME-13-0-s005.pdf]

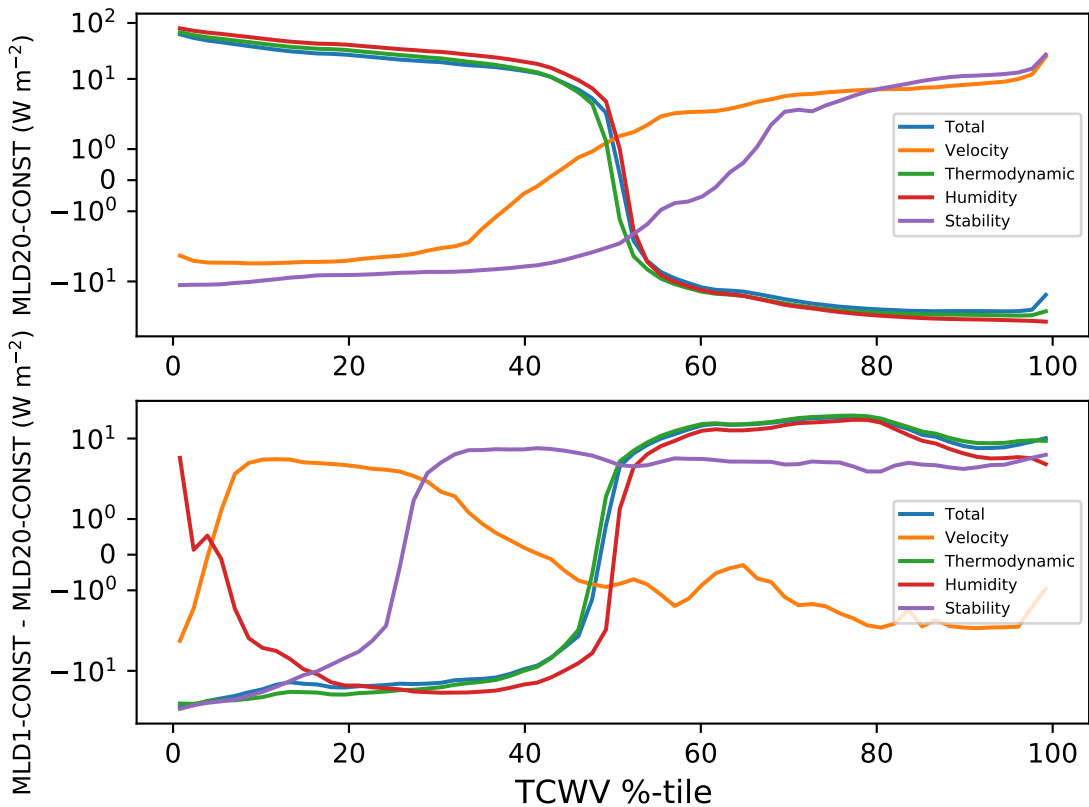

Supplement: Supplementary file 4 — Figure S3 [file JAME-13-0-s001.pdf]

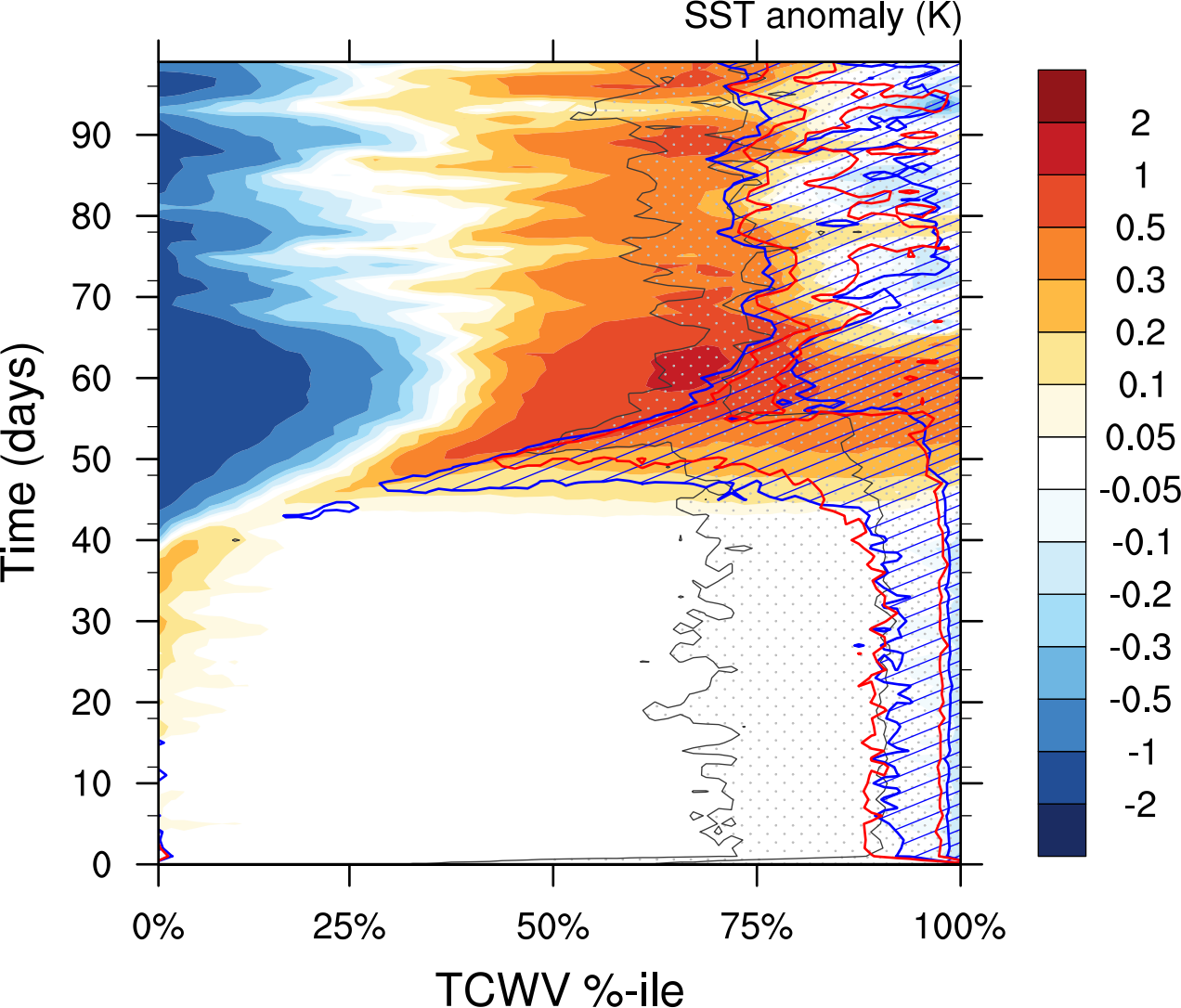

Supplement: Supplementary file 5 — Figure S4 [file JAME-13-0-s002.pdf]

(a) MLD1-CONST

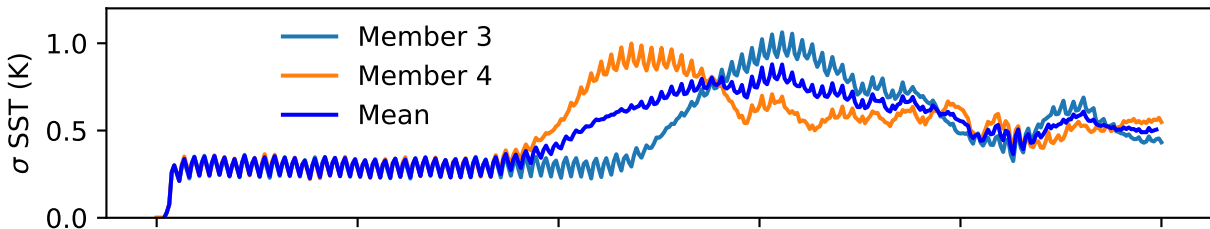

(b) MLD1-DIURN

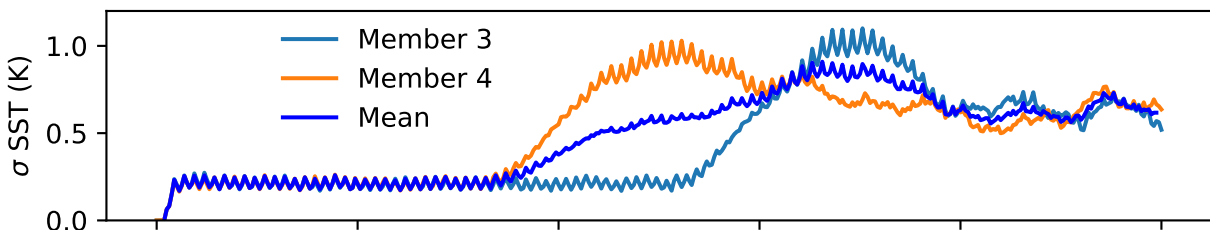

(c)

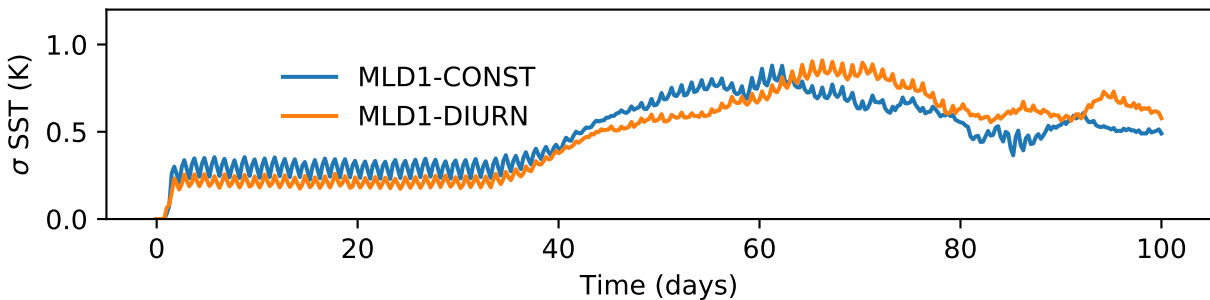

Supplement: Supplementary file 6 — Figure S5 [file JAME-13-0-s004.pdf]

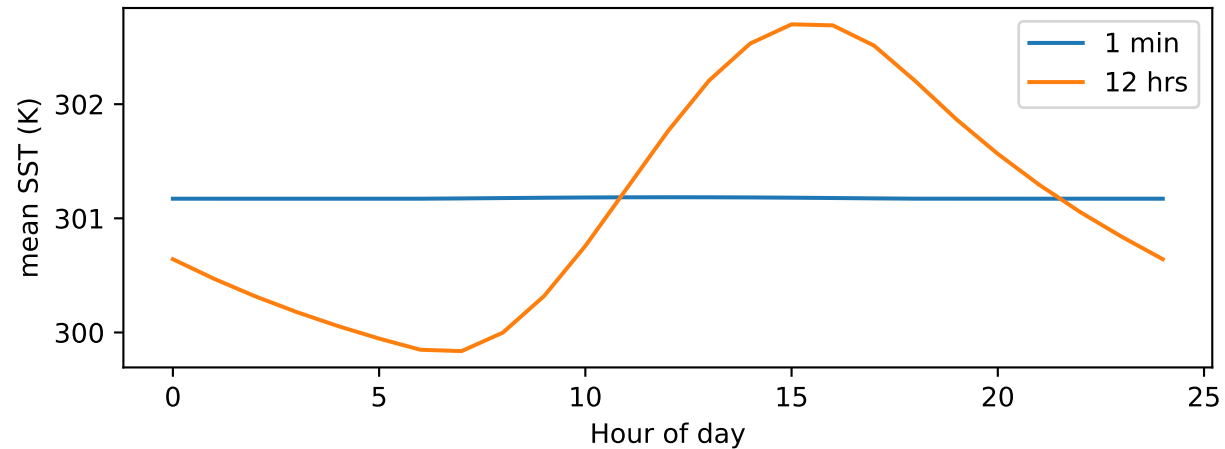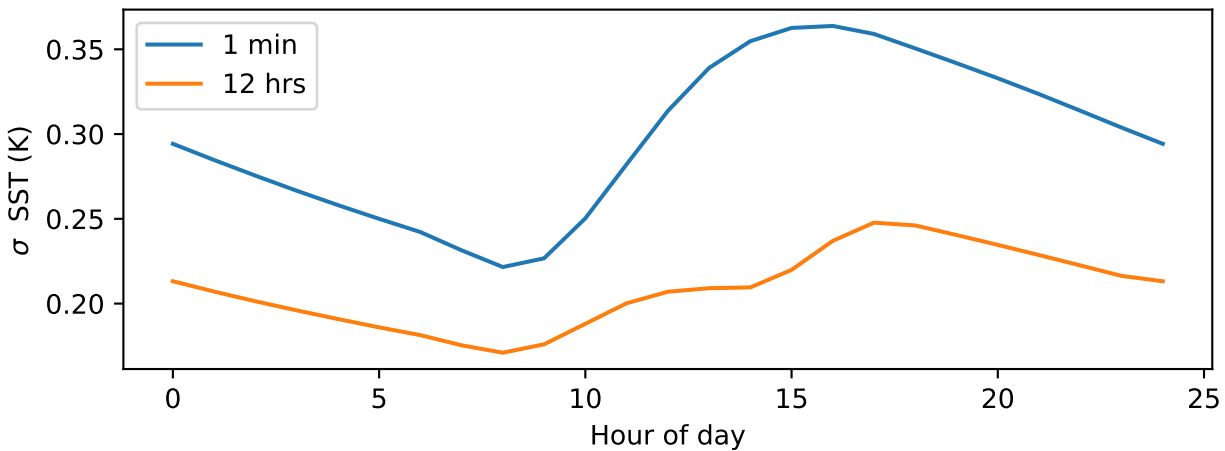

Supplement: Supplementary file 7 — Figure S6 [file JAME-13-0-s007.pdf]
